# GeenaR: A Web Tool for Reproducible MALDI-TOF Analysis

**DOI:** 10.3389/fgene.2021.635814

**Published:** 2021-03-29

**Authors:** Eugenio Del Prete, Angelo Facchiano, Aldo Profumo, Claudia Angelini, Paolo Romano

**Affiliations:** ^1^Institute for Applied Mathematics, National Research Council, Naples, Italy; ^2^Institute of Food Sciences, National Research Council, Avellino, Italy; ^3^Proteomica e Spettrometria di Massa, IRCCS Ospedale Policlinico San Martino IST, Genova, Italy

**Keywords:** mass spectrometry, proteomics, cancer analysis, reproducible research, web tool

## Abstract

Mass spectrometry is a widely applied technology with a strong impact in the proteomics field. MALDI-TOF is a combined technology in mass spectrometry with many applications in characterizing biological samples from different sources, such as the identification of cancer biomarkers, the detection of food frauds, the identification of doping substances in athletes’ fluids, and so on. The massive quantity of data, in the form of mass spectra, are often biased and altered by different sources of noise. Therefore, extracting the most relevant features that characterize the samples is often challenging and requires combining several computational methods. Here, we present GeenaR, a novel web tool that provides a complete workflow for pre-processing, analyzing, visualizing, and comparing MALDI-TOF mass spectra. GeenaR is user-friendly, provides many different functionalities for the analysis of the mass spectra, and supports reproducible research since it produces a human-readable report that contains function parameters, results, and the code used for processing the mass spectra. First, we illustrate the features available in GeenaR. Then, we describe its internal structure. Finally, we prove its capabilities in analyzing oncological datasets by presenting two case studies related to ovarian cancer and colorectal cancer. GeenaR is available at *http://proteomics.hsanmartino.it/geenar/*.

## Introduction

Mass spectrometry (MS) is the experimental technology widely applied in proteomics studies to reveal signals of peptides, proteins, and other molecules in samples from various sources (Boersema et al., 2015). In the last years, researchers carried on many proteomics studies, with an increasing interest in upcoming results: nowadays, data from MS technologies are an essential resource for proteomics analysis (Wagner et al., 2003; Han et al., 2008; Li and Tang, 2016). The MS analysis of a mixture of proteins or peptides generates a spectrum of mass/charge signals representing the sample proteomic profile. The computational analysis of a large number of samples may lead to their classification, based on the profile’s features, or to identify marker signals, considered as fingerprints for several conditions. Many studies in the biomedical area take advantage of proteomics and MS data, looking for information useful to diagnostic, classification, or novel biomarkers discovery of a pathological state under investigation (Mazzeo et al., 2008; Liu and Ouyang, 2013; Prieto et al., 2014).

One of the most common MS technologies used in proteomics is named MALDI-TOF (Matrix-Assisted Laser Desorption and Ionization Time-Of-Flight). This technology concerns proteins of a sample that are co-crystallized with compounds suitable for absorbing UV radiations: when a UV laser beam hits the crystal, energy absorbed by the compounds vaporizes the crystals, and the proteins are ionized, desorpted and then addressed to the MS analysis. SELDI-TOF (Surface-Enhanced Laser Desorption and Ionization Time-Of-Flight) is a modified version of the MALDI-TOF technology, also used for biomarkers discovery (Cotter, 1998; Greco et al., 2018). The output from a spectrometer is a set of raw mass spectra from different samples. Usually, the scientist collects one mass spectrum per sample (or more mass spectra per sample, if technical replicates are present). Mass spectra are affected with disturbs, such as not constant variance of noise, spike noise, background noise or batch noise, which need robust pre-processing steps before a more in-depth analysis (Coombes et al., 2007).

As for other omics technologies, MS produces a high volume of experimental data. Many online repositories in the proteomics field, such as the best-known *PRoteomics IDEntification database* (*PRIDE*) (Perez-Riverol et al., 2019) or *ProteomicsDB* (Samaras et al., 2020) are fundamental to let worldwide researchers retrieve datasets for their studies. The *ProteomeXchange* consortium (Vizcaíno et al., 2014; Deutsch et al., 2017) is active in the standardization of data submission and the dissemination of mass spectrometry proteomics data, guiding the researchers to download robust proteomic datasets.

The extraction of the most relevant features that characterize the samples is still challenging and requires combining several computational methods. Consequently, mass spectra processing and analysis is an active field of investigation, with novel tools continuously developed (Basharat et al., 2019; Chen et al., 2019; Bouyssié et al., 2020). In particular, the tools for analyzing these data require not only to improve their performances and adapt their application to the changing technology but also enhance the usability and the reproducibility of results. Two well-known software packages for analyzing proteomics data are *MaxQuant* (Tyanova et al., 2016) and *OpenMS* (Pfeuffer et al., 2017). Both tools are freely available, together with the manuals. *MaxQuant* is specialized in high-resolution MS data, with many labeling techniques, label-free quantification methods, a viewer application for the visualization of raw mass spectra and results, and the possibility of a framework for the statistical analysis of the output. *OpenMS* is a versatile open-source library for mass spectrometry data analysis, with workflows usable by command-line or integrated on a platform, comprehensive of viewer application and report capabilities. Overall, *MaxQuant* and *OpenMS* are complete tools with a large number of functionalities. However, their usage requires experienced users and suitable computational resources.

The reproducibility and transparency of the computational analysis of biological experiments are an essential part of the research process to assess and validate the findings and compare them with results obtained under different conditions or by applying other methods and parameters. Unfortunately, several publications with omic data analysis are (at least partially) false or not entirely reproducible, as reported in Ioannidis (2005), due to poorly described computational protocols. Therefore, the scientific community has underlined the importance of adopting reproducible research standards when analyzing high-throughput omics data (Russo et al., 2016a; Brito et al., 2020). The work of Sandve et al. (2013) provides golden rules to obtain a (computationally) reproducible research. The main idea is to incorporate data, user parameters and results in a human-readable document built under the principles of literate statistical programming (Peng, 2011). While such approaches are becoming popular in statistics, their use within web-tools or graphical user-friendly interfaces is still challenging, with few exceptions (Russo et al., 2016b).

In the past years, we developed Geena and its evolution in Geena 2, a tool for managing MALDI-TOF mass spectra (Romano et al., 2016, 2018), to offer a user-friendly tool useful for filtering, averaging different volumes of data, and comparing them by mass spectra alignment. Our group and other researchers adopted these tools for the differential analysis of peptidomes in oncological studies (Profumo et al., 2013; Boccardo et al., 2015; Sun et al., 2017; Standke et al., 2019). Starting from the architecture of Geena 2 and the preliminary results obtained in Del Prete et al. (2016), here we present GeenaR, an original user-friendly web tool, available online, based on the R environment and conceived for the automation of different tasks in MALDI-TOF mass spectra analysis. GeenaR provides the possibility to handle several file formats for the mass spectra, offers a wide range of statistical methods for pre-processing mass spectra, and visualizes results in a graphical form. Moreover, GeenaR also produces a human-readable report that contains the choice of the function parameters, the executed steps, the results, and the code used for processing the mass spectra. Above all, GeenaR is user-friendly, and its usage does not require any computational language knowledge. In this work, we illustrate the workflow available in GeenaR. Then, we describe its internal structure. Finally, we demonstrate the capabilities of GeenaR by presenting the results of two case studies taken from literature, referring to ovarian cancer and colorectal cancer datasets.

## Materials and Methods

### GeenaR Workflow

**FIGURE 1:**
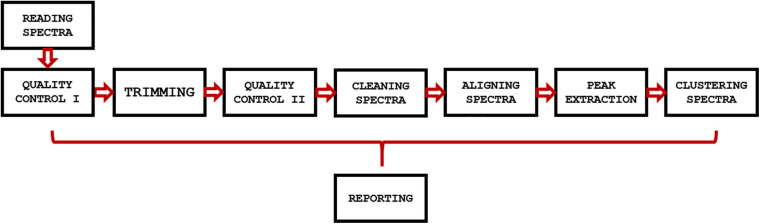
GeenaR workflow. The workflow implemented in GeenaR consists of several modules that are executed in cascade. Each module performs one or more tasks and transfers the intermediate results to the subsequent module. The reporting module collects results from the other modules.

GeenaR is a web-tool that provides a complete workflow for the analysis of MALDI-TOF mass spectra. The user can upload the mass spectra files, select the steps-methods to perform on the dataset, and obtain all the plots of mass spectra (raw and processed) and other graphical results, together with a resume report with the results and the R code (if selected). In other words, GeenaR allows a user to execute a pipeline consisting of different modules. Each module performs one or more tasks. Figure 1 illustrates the workflow implemented in R.

GeenaR user interface imports the mass spectra and the target file that describes the mass spectra metadata (filename, sample, replicate, and group). Then, it imports the job name/dataset name, the user’s choices for the modules that have to be executed, and the parameters selection. After that, it loads the necessary libraries and all the modules for the step-by-step processing. Then, it calls the required modules until the end of the workflow. After completing a module, it stores the results in pre-organized folders using pre-established file formats. Finally, it returns the results to the user interface. We describe each module below.

#### The Reading Spectra Module

This module allows reading the raw mass spectra and the target file that describes the experimental design. Then, it creates the R object that GeenaR will use during the analysis. Data and meta-data are internally stored in the *MassSpectrum* class format. The target file with information on mass spectra is a text file with the name and extension of the files, samples, replicates, and groups (if available).

#### The Quality Control Module

This module performs a preemptive exploratory analysis of the raw mass spectra and allows identifying potential outliers. It also plots the raw mass spectra and stores the files in a subfolder that the user can retrieve at the end of the analysis. This module also provides a log file with a summary of the processed files. The log file reports the following information:

– the methods and parameters selected by the user, as taken from the attributes file of the process;
– the list of the mass spectra as taken from the folder with the mass spectra files, with the information about samples and replicates;
– the numerosity of m/z values for each mass spectra (associated with the resolution);
– the range of m/z values for each mass spectra (i.e., minimum and maximum values);
– the range of the intensity values for each mass spectrum (i.e., minimum and maximum values);
– the possible presence of empty mass spectra;
– the possible presence of resolution irregularity (i.e., irregular frequency of m/z values in intervals, compared to a fixed threshold).

GeenaR identifies potential mass spectra outliers using the *atypicality score* (A score), defined as the Rousseeuw’s Q value normalized to the median intensity of the raw mass spectrum (Hedges, 2008). In particular, GeenaR suggests the mass spectra with an A score above an upper bound or below a lower bound as potential outliers. However, it does not remove them from the analysis: the choice of eliminating the mass spectra from the data set under analysis is left to the user. Quality control is executed both before and after the mass spectra trimming when requested to verify if the trimming modifies, reduces, or eliminates the potential outliers.

#### The Trimming Spectra Module

This module allows selecting a pre-specified range of m/z values from the raw mass spectra. Then, it plots all the trimmed mass spectra and stores them in a subfolder for the retrieval at the end of the analysis. The user can specify the trimming range by fixing the lower and higher m/z values.

#### The Cleaning Spectra Module

This module completes multiple consecutive tasks for the adjustment of the mass spectra. More in detail:

– *Variance Stabilization*. This task applies a transformation on the mass spectra intensities to cope with possible very high values and reduce the dependency between variance and mean value. *Square root transformation* and *log transformation* (e-, 2-, and 10- base) are available (Välikangas et al., 2018);
– *Smoothing*. This task smooths the mass spectra to reduce possible spikes that are close to each other (spike noise), improving the profile of the signal. The available smoothing filters are *Savitzky-Golay* (Fredriksson et al., 2007) and *Moving Average* (Mo et al., 2010). Both filters need the user to specify the window size, i.e., the number of m/z values to be included as the local range to use;
– *Baseline Correction*. This task corrects the mass intensities to remove possible differences in the signal coming from changes or interferences in the experimental condition (background noise), which may alter the base level of the mass spectra. The baseline correction methods available in GeenaR are *Statistics-sensitive Non-linear Iterative Peak-clippin*g (SNIP) (Ryan et al., 1988), *Top Hat* (van Herk, 1992), *Convex Hull* (Andrew, 1979), and *median* (Gil and Werman, 1996). SNIP method requires the user to specify the number of iterations, whilst Top Hat method requires the user to specify the window size. GeenaR removes the estimated value of the baseline to all the mass spectra;
– *Normalization*. This task normalizes the mass spectra intensities to overcome differences due to mass spectra acquisition times. Indeed, the spectrometers need frequent calibrations, and results may have slight changes between different calibrations (batch noise). The available normalization methods are *Total Ion Current* (TIC), *Probabilistic Quotient Normalization* (PQN) (Dieterle et al., 2006), and *median*.

GeenaR plots all the transformed, smoothed, corrected, and normalized mass spectra and stores them in the corresponding subfolders that the user can retrieve at the end of the analysis.

#### The Aligning Spectra Module

This module executes the tasks of averaging, aligning, and plotting processed mass spectra. If any replicates are present for one or more samples, GeenaR computes an average mass spectrum from all the replicate mass spectra of a single sample representative of all the replicates. The user should specify if desires to perform this task and the method to use (*sum, mean, or median*). Then, GeenaR aligns all the mass spectra (averaged or not) and calibrates them (phase correction) using one of the following functions: *lowess, linear, quadratic, and cubic*. Furthermore, it calculates an overall estimation of the noise using the *Median Absolute Deviation* (MAD) or the *Super Smoother* algorithm (Friedman, 1984), after the selection of the signal-to-noise (SNR) values, the window size, and the tolerance value for the resolution inside the window size. GeenaR creates two subfolders in the presence of replicates, one for the plots of all the averaged mass spectra and one for the plots of all the aligned mass spectra; if replicates are absent, GeenaR creates only the second subfolder.

#### The Peak Extraction Module

This module executes various consecutive tasks to identify the most relevant peaks for each mass spectrum and the entire dataset. More in details:

– *Peak Detection*. GeenaR defines a peak as a local maximum of the mass spectra. In this step, peaks are identified for each mass spectrum (or averaged mass spectrum). GeenaR inherits the algorithm, *MAD* or *Super Smoother*, and the related parameters, the window size and the SNR, from the aligning task;
– *Peak Binning*. Since peak positions might be very similar (but not identical) after the alignment, GeenaR performs a binning step. In this step, peaks in the different mass spectra are assigned to the same m/z value by considering a tolerance value. The binning method concerns the concept of strict and relaxed bins, respectively, when all the peaks or just the highest ones are selected;
– *Peak Filtering*. The user can control the occurrence of peaks over all the mass spectra in terms of percentage. The coverage parameter defines the percentage of samples supporting the peaks, acting as a trade-off between variance and bias, and globally controlling the number of significant peaks.

GeenaR generates the feature matrix (peak matrix) with the peaks list, where the columns represent the m/z values of the most important peaks, and the rows represent the intensities of the peaks for each sample. Furthermore, the feature matrix is provided as one of the results of the analysis. Therefore, the user could use it with their favorite methods available in another computational environment. Moreover, GeenaR depicts the feature matrix in the form of a heatmap, to show the peak distribution for the mass spectra, and stores the plots of the peaks for each mass spectrum in the corresponding subfolder, providing a series of statistical methods for its analysis.

#### The Clustering Spectra Module

This module processes the feature matrix and allows carrying out different tasks for inspecting sample profiles and clustering the mass spectra. As the first step, GeenaR performs the Principal Component Analysis (PCA) on the feature matrix (Shao et al., 2012) and displays the results by projecting the samples in the first three principal components space. This projection allows exploring the data and identifying similarities among samples. Then, GeenaR computes the similarity matrix using the pairwise cosine correlation as a similarity measure. The conversion in a distance matrix allows the creation of a dendrogram for the mass spectra, and the linkage methods available are w*ard, complete linkage, average linkage*, or *Gower’s median*, respectively. If the user does not suggest an expected number of clusters, GeenaR provides either the gap statistic or silhouette methods for estimating this number.

#### The Reporting Module

The analysis performed using GeenaR is fully reproducible since at the end of the analysis it is possible to obtain a human-readable report that includes all the steps that the user performed. In particular, the report is in .html format with information about the R packages used in the GeenaR workflow, the values of the parameters selected by the user, the names of the uploaded files, the results from quality control, and the plots generated for the heatmap, the estimate of clusters number, and cluster dendrogram. Furthermore, the user can download the log file, the feature matrix, and all the mass spectra plots (raw, trimmed, stabilized, smoothed, corrected, normalized, averaged, aligned, and peaks) in a compressed format, from links at the end of the report. Finally, for transparency, GeenaR generates a version of the report with all the R code processed by the pipeline (without the embedded mass spectra), thus the user can reproduce the results and apply the workflow with different methods/parameters, or with other datasets.

### Web User’s Interface

**FIGURE 2:**
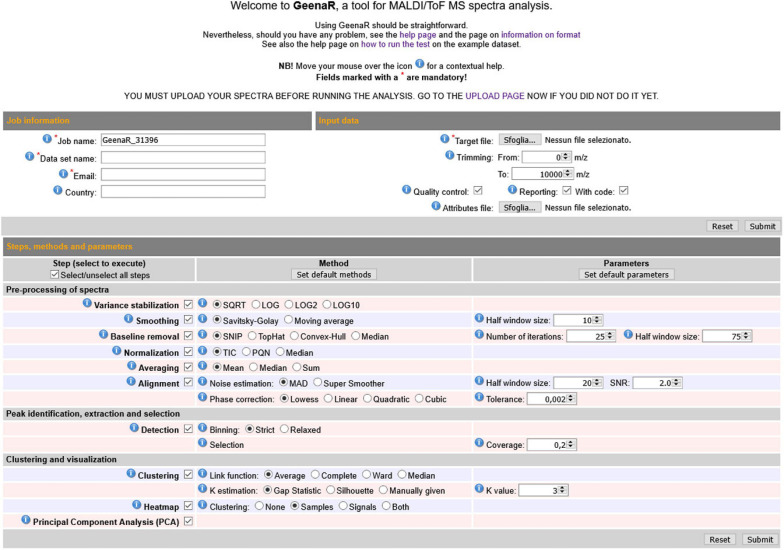
GeenaR web user’s interface. The web user’s interface of GeenaR consists of the following sections: Job Information, Input Data, Steps-Methods-Parameters. By selecting suitable steps, methods, and parameters, the user can customize the analysis of a given dataset. For ease of usage, most of the elements present default values. Moreover, the interface provides contextual help by moving the mouse over an icon.

The user-friendly web interface is divided into three main sections: “Job information,” “Input data,” “Steps, methods and parameters,” as shown in Figure 2. The user should compile the requested fields, then press the *Submit* button and wait to visualize the results as a human-readable report with links to data and figures.

The “Job information” section captures the job name to identify the analysis, the name of the dataset, an email for any contact about the work evolution, and the user’s country for a simple statistical purpose. The email address must be the same used at uploading time, when the dataset was submitted to the server.

The “Input data” section allows uploading the target file (i.e., a .txt file with the mass spectra file names along with the metadata on samples, replicates, and groups). In this section, the user can also upload a precompiled attributes file that configures the steps and parameters of the analysis to execute. Alternatively, the user can select the desired attributes by filling in the “Steps, methods, and parameters” section. Moreover, in the same section, the user can choose the trimming range and select the quality control and the reporting steps.

The “Steps methods and parameters” section consists of three vertical subpanels denoted *Step, Method*, and *Parameters*. The subpanel *Step* is divided into three main parts: (1) pre-processing of mass spectra, (2) peak identification, extraction, and selection, (3) clustering and visualization. The subpanel *Methods* allows selecting the desired algorithms/functions for the execution of tasks. The subpanel *Parameters* allows defining numerical values that are required for the previous methods. The user can select (check) methods in the *Methods* subsection and write values in the *Parameters* subpanel. Table 1 reports all the tasks, steps, methods, and parameters available, both automatic and user-selectable. GeenaR proposes default values for most of the cases.

**TABLE 1:** List of tasks, steps, methods, and parameters of GeenaR web user’s interface.

**Task**	**Step**	**Method**	**Parameters**
Reading	Import files		
	Read target file		
	Acquire metadata		

Quality control	Create log file		
	Detection of outliers		
	Plot raw spectra		

Trimming	Trim raw spectra		Min-Max, User
	Plot trimmed spectra		

Cleaning	Variance stabilization	sqrt, logE, log2, log10	
	Plot stabilized spectra		
	Smoothing	Savitzky-Golay, Moving Average	Half window size
	Plot smoothed spectra		
	Baseline correction	SNIP	Number of iterations
		Top Hat	Half window size
		Convex hull, median	
	Plot corrected spectra		
	Normalization	TIC, PQN, median	
	Plot normalized spectra		

Averaging and aligning	Average replicates	Mean, median, sum	
	Plot averaged spectra		
	Align samples	a. MAD, Super Smoother	Half window size, SNR
		b. Lowess, linear, quadratic, cubic (*)	Tolerance
	Plot aligned spectra		

Peak extraction	Peak detection	MAD, Super Smoother	Half window size, SNR
	Peak binning	Strict, relaxed	Tolerance
	Peak filtering		Minimum frequency
	Create feature matrix		
	Create heatmap		
	Plot peaks		

Clustering and visualization	PCA		
	Plot PCs and top loadings		
	Estimate number of clusters	Gap statistic, silhouette	User
	Clustering	Ward, complete, average, median	
	Plot clusters estimation		
	Plot dendrogram		

Reporting	Generate html report with spectra		
	Generate html report with R code		

The tasks are in the first column, the steps are in the second column, the methods are in the third column, and the parameters are in the fourth column. Rows indicate methods and parameters related to the corresponding steps. Selectable steps-methods are slightly different from the web page. (*) Two different kinds of methods for the same step.

The GeenaR web user’s interface also contains (a) an upload page to submit mass spectra to the server; (b) a help page with all the information necessary for the user to understand how GeenaR works and how to select methods and parameters for the analysis of the mass spectra; (c) an information page, with details on the mass spectrum formats that GeenaR can handle, and how the user should provide the target and attributes files (see Project Links).

The upload page is especially useful since it allows the user to upload data once and then analyze them many times, with various parameters, thus significantly reducing the overall execution time. Users can upload the mass spectra of a dataset by submitting a .zip compressed file (allowed mass spectra file formats are those accepted by the *MALDIquantForeign* R package). The dataset name and the email address are used jointly in order to define for each dataset a unique folder, from which mass spectra are retrieved at the execution time. Mass spectra can be incrementally added into the folder, thus allowing uploading of subsets of the same dataset at different times. Users can then make reference to mass spectra in a given dataset if and only if they know all related information: mass spectra file name, dataset name and email address. Datasets incidentally sharing the same name do not overlap, unless they are linked to the same email address.

### Type of Data: Input and Output

To start the analysis with GeenaR, the user has to provide (1) a target file (a .txt file) with the list of MALDI-TOF mass spectra and the metadata of the experimental design and (2) the set of mass spectra that he/she wants to analyze. Optionally the user can provide an attributes file that contains the list of steps, methods, and parameters to apply to the mass spectra dataset. If not provided, GeenaR generates the attributes file using the user choices in the section “Steps, methods and parameters.” The information page includes a detailed description and an example of how to organize and format both the target file and the attributes file.

Usually, MALDI-TOF mass spectra consist of two columns: the first column represents the m/z value (*x*-axis), and the second represents the intensity (*y*-axis). Therefore, each couple m/z value-intensity depicts a point, and all the points plot a mass spectrum, where higher intensity values are considered peaks. A set of peaks can represent the entire mass spectrum. GeenaR is able to read many file formats for the MALDI-TOF mass spectra (.txt, .tab, .csv, .fid, .ciphergen, .mzXML, .mzML, (Deutsch, 2010), imzML, .analyze, .cdf, .msd) with an automatic detection.

GeenaR provides the name of the main folder (job name) and all the steps, methods, and parameters to the analysis layer (see Overall Structure description) in the form of a .csv file (attributes file), and creates two subfolders: *Spectra*, where it stores all the mass spectra for temporary usage, and *Results*, where it writes all the files generated from the workflow during its execution.

**FIGURE 3:**
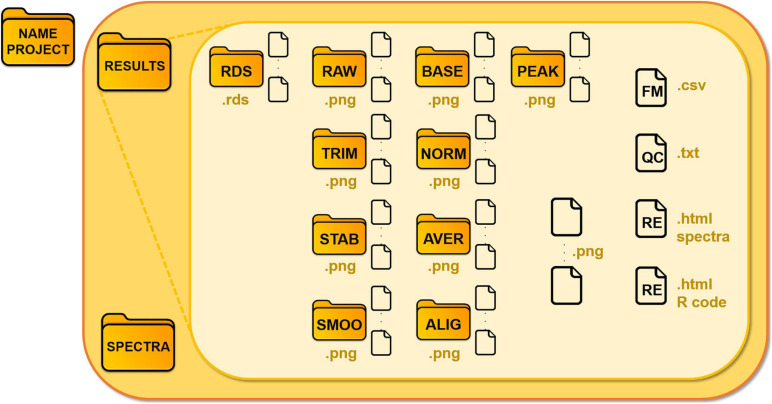
Structure of the data folder of a project. GeenaR associates the analysis of each dataset with a project and a folder, where it locates *Results* and *Spectra* subfolders, respectively. Subfolder *Spectra* contains the uploaded mass spectra, subfolder *Results* is organized in different folders according to the steps of the analysis. The folders are: RDS for the R object files; RAW for the raw mass spectra; TRIM, for the trimmed mass spectra; STAB for the stabilized mass spectra; SMOO for the smoothed mass spectra; BASE for the corrected mass spectra; NORM for the normalized mass spectra; AVER for the averaged mass spectra (when replicates are available); ALIG for the aligned mass spectra (for samples); PEAK, for the peaks from mass spectra. Other files: FM, feature matrix; QC, quality control; RE, report.

We illustrate the schema of the main folder in Figure 3. Each module saves .rds files (R object files), which are requested as input for the following modules, in the “Results/Rds” subfolder. GeenaR stores all the mass spectra, from raw to peak, in devoted subfolders, as .png graphic files.

Moreover:

• the plots of the graphic results (quality control (QC), PCA, heatmap, gap statistic/silhouette, dendrogram) are saved as .png graphic files;
• the feature matrix (FM) is a .csv file;
• the analysis reports (RE) are in .html readable from a web browser.

Note that GeenaR embeds the log file, the feature matrix and the processed mass spectra plots in the .html report files. There are two report files: (i) a simple document that includes the descriptions of the steps that GeenaR executed, the parameters used and a selection of plots, (ii) a detailed document that also incorporates the R code used for processing the spectra. The second report is especially suited for expert users which are familiar with the R language and want to inspect and reproduce the findings in a transparent way. Both reports contain links to the log file, the feature matrix and the entire set of figures and data produced by GeenaR The user can download the corresponding files by clicking on the links: a. csv file for the feature matrix and a .zip file for each set of figures.

At the end of the analysis, GeenaR provides an output page including: (a) *job summary section*, with the information on the reference dataset and the uploaded target file, the generated attributes file (which can be downloaded for later reuse), the steps and methods performed during the job; (b) *elaboration section*, that is filled with detailed information on the ongoing elaboration and the timeline of each step, and links to download the feature matrix and the reports at the end of the analysis; (c) *results section*, including some essential plots generated by GeenaR. Furthermore, GeenaR sends a summary of the job with links to results to the user, by email.

### Overall Structure

Starting from the background structure of Geena2, we developed GeenaR, an integrated web tool that allows the user to pre-process and analyze MALDI-TOF mass spectra. GeenaR is based on the Linux-Apache-MySQL-PHP (LAMP) environment, a well-known open-source web service stack, and integrates it with the R programming language and environment (R Core Team, 2020). We already tested the efficiency and stability of LAMP with Geena2. Here, we choose to develop the statistical core using the R environment for its portability and the availability of several statistical analysis methods. Conceptually, the architecture consists of three layers inside the LAMP system, as shown in Supplementary Figure 1:

1. the analysis layer, in which different cascade modules (i.e., scripts) in R language perform all the methods for pre-processing and analyzing data, plotting and storing results;
2. the interconnection layer, in which a PHP script collects the choice of the user, prepares the execution environment by also providing all parameters to the analysis layer, monitors the execution, and provides access to results;
3. the web user-interface, in the HTML and Javascript languages, that facilitates the selection of the methods and parameters by the user and passes them to the interconnection layer.

### R Packages

We implemented the statistical core of GeenaR using the R language and wrapping several existing R packages, available in Bioconductor (Huber et al., 2015) or Comprehensive R Archive Network (CRAN) repositories. GeenaR executes several modules in cascade, where each module corresponds to a specific script. The modularity of this approach allows inserting new functionalities without rewriting all the code.

Different R packages (libraries) perform the above mentioned tasks: *MALDIquantForeign* enables the acquisition of the MALDI-TOF mass spectra, with the automatic recognition of the uploaded file type (Gibb, 2019); *MALDIquant* empowers the creation of object classes for the treatment of the mass spectra, a simple quality control, the trimming, the cleaning, the averaging, the alignment, the peak extraction, and the creation of the feature matrix (Gibb and Strimmer, 2012); *MALDIrppa* allows the estimate of possible mass spectra that can be outliers for the entire dataset (Palarea-Albaladejo et al., 2018); *cluster* incorporates the application of the gap statistic method for the estimation of the number of possible groups for the mass spectra (Maechler et al., 2019); *lsa* allows the calculation of the pairwise cosine correlation between the list of peaks from each mass spectrum, with the subsequent creation of the similarity matrix (Wild, 2020); *dendextend* allows a better dendrogram of the mass spectra (Galili, 2015); *mixOmics* allows computing PCA and plotting PC figures (Rohart et al., 2017); *pheatmap* allows creating fancy heatmap, with many parameters under user’s control (Kolde, 2019); *rmarkdown* enables the rendering of the report, written with *roxygen* comments for documenting the code (Xie et al., 2018; Allaire et al., 2020); *kableExtra* defines fancy tables for the reporting (Zhu, 2019).

### Project Links

GeenaR web tool is available at the following link: *http://proteomics.hsanmartino.it/geenar/*. Datasets can be uploaded from the upload page at *http://proteomics.hsanmartino.it/geenar/upload.php.* The help page is available at *http://proteomics.hsanmartino.it/geenar/help.php* and the page with information on the format of files is available at *http://proteomics.hsanmartino.it/geenar/info.php*.

## Results

Considering our previous experiences in the oncology domain for the selection of the datasets to use as case studies, we illustrate the capabilities of GeenaR in analyzing mass spectra data using two case studies from two different typologies of tumor: one dataset concerns an ovarian cancer (case study 1) and one dataset concerns a colorectal cancer (case study 2).

### Case Study 1: Ovarian Cancer

Low molecular weight serum protein patterns can help to determine the pathological state of the organs, allowing the detection of cancer in individuals. In an original study, researchers analyzed sera for studying the difference between women with ovarian cancer and healthy controls. They provided evidence for the use of a proteomic pattern to screen all the stages of ovarian cancer, both in high-risk and general subjects. More details are reported in Petricoin et al. (2002).

The dataset consisted of 200 mass spectra divided into four different groups of the same size: 50 samples with ovarian cancer patients constituting the groups A-B and 50 healthy individuals forming the C-D groups (control groups). Mass spectra were generated by the Surface-Enhanced Laser Desorption and Ionization Time-Of-Flight (SELDI-TOF) mass spectroscopy technique, a derived technology of MALDI-TOF which couples it with a selective analyte capture mechanism, and produces classical proteomic patterns (as explained in Overall Structure section). Each mass spectrum consisted of around 15,200 values, in the range 0–20,000 m/z. In this illustrative example, we jointly analyzed with GeenaR the mass spectra extracted from *Clinical Proteomics Program Databank—Proteomic Patterns*, low-resolution SELDI-TOF study sets, A. Ovarian Cancer Studies, 2. Data from unpublished experimental studies, i. 4/3/02 Ovarian Study set (repository link: https://home.ccr.cancer.gov/ncifdaproteomics/ppatterns.asp, dataset link: https://home.ccr.cancer.gov/ncifdaproteomics/OvarianDataset4-3-02.zip, subfolders *Cancer* and *Control*).

After compiling a target file reporting the file names and the group of belonging for each mass spectrum, we executed GeenaR with the following choices:

A. we selected all the steps, skipping the average step;
B. we trimmed the raw mass spectra in the range 0–12,000 m/z;
C. we used the square root method for stabilization, Savitzky-Golay method with a half window of 10 points for smoothing, SNIP method with 25 iterations for baseline correction, TIC method for normalization;
D. we chose the MAD method with a half window size of 20 points, 2 as SNR, tolerance of 0.002 for the noise estimation, and the lowess method for the phase correction, both in alignment step;
E. we selected the strict method for peak binning and 50% of coverage for peak selection;
F. we did not apply any clustering algorithm when plotting the heatmap;
G. we used the average method as the link function for clustering, with *k* = 4.

**FIGURE 4:**
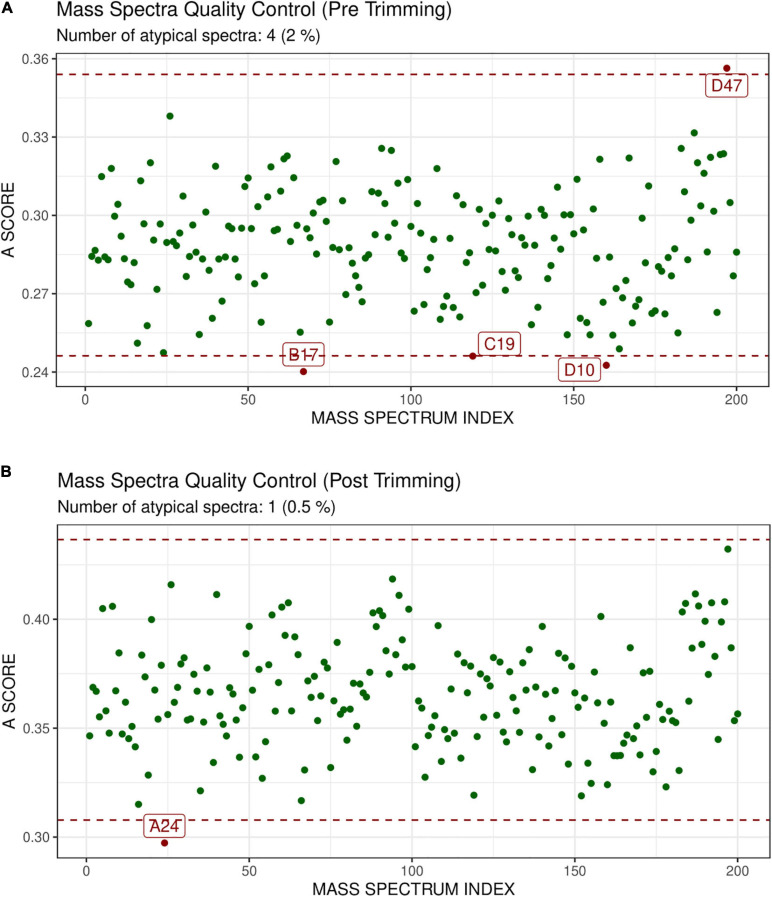
Atypicality score plot before trimming **(A)** and after trimming **(B)** for Case Study 1 (ovarian cancer). Each green point represents a sample. The red dotted lines represent the lower and upper values for the A-score. Samples with an A-score larger than the upper value or smaller than the lower value could be potential outliers. They are colored in red and enlighten with their names. **(A)** Shows the samples before trimming, where D47, B17, C19, D10 are identified as possible outliers. **(B)** Shows the case after trimming the mass spectra in the m/z range 0–12,000. The mass spectrum signed as A24 is a possible outlier.

GeenaR did not detect any empty mass spectra. The quality control pre-trimming identified as possible outliers the samples D47, B17, C19, D10, as depicted in Figure 4A. The number of potential outliers represented 2% of the mass spectra (4 out of 200). For illustrative purposes, here we trimmed the spectra in the m/z range of 0–12,000. The quality control post-trimming step showed how cutting the noise toward the tail can improve the study and decrease the number of outliers. In this case, there was only one outlier, sample A24, as depicted in Figure 4B. So, the percentage of potential outliers reduced to 0.5% of the mass spectra (1 out of 200).

**FIGURE 5:**
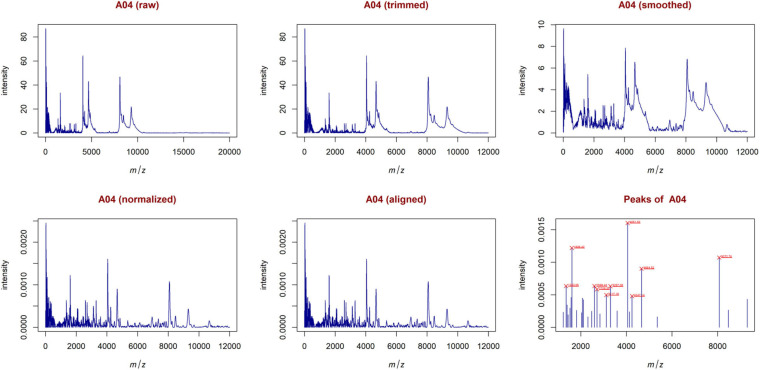
Examples of plots created during the entire analysis for Case Study 1 (ovarian cancer). For each sample, GeenaR produces a series of visualizations of the mass spectra as intensity versus m/z from raw, trimmed, smoothed, normalized, and aligned spectra, and so on. GeenaR also plots the most relevant peaks with the 10 highest peaks enlighten in red: 1353.05, 1606.42, 2598.43, 2708.53, 3117.18, 3297.08, 4051.53, 4247.14, 4664.52, 8072.76. The panels show an illustrative example with sample A04.

From the trimmed samples, GeenaR extracted a feature matrix with the 200 mass spectra and 31 relevant peaks, most of them present in several samples. Figure 5 shows the spectrum of sample A04 (m/z versus intensity) as raw, trimmed, smoothed, normalized, and aligned, respectively. Moreover, it also shows the list of relevant peaks identified in the sample. The difference between the number of peaks identified for sample A04 (i.e., 27) and the peaks present in the feature matrix (31) is due to the absence of some peaks in the given sample.

From the feature matrix, GeenaR created the heatmap shown in Supplementary Figure 2 that allows identifying which peaks are essential for some mass spectra visually. It is possible to notice that the most informative peaks were: 1226.0592, 1573.8132, 1606.4189, 4051.5320, 4664.5196, 8072.7649. More in detail, 1573.8132 and 1606.4189 had an inverse trend for group C and part of group D, and 4051.5320 was a little more relevant for groups A-B.

**FIGURE 6:**
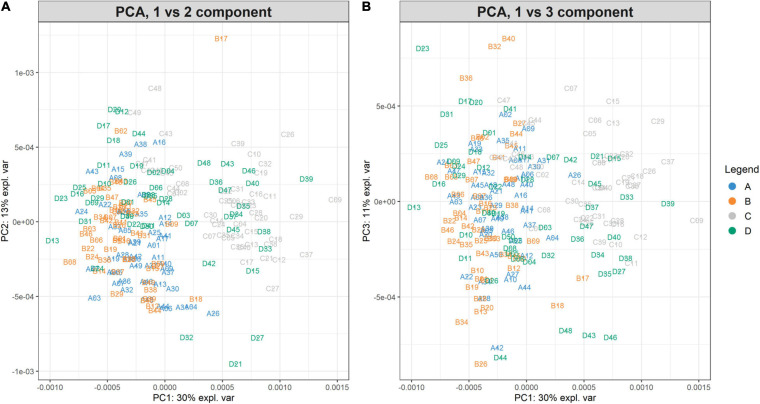
PC1vsPC2 and PC1vsPC3 plots for Case Study 1 (ovarian cancer). The PCA analysis of the feature matrix revealed that PC1, PC2, PC3 accounted for 54% of the total explained variance. **(A)** Shows the projection in the PC1 versus PC2 space, **(B)** the projection in the PC1 versus PC3 space. Samples are colored according to their group. Ovarian cancer groups (A, B) are overlapping, whilst control groups (C, D) are not clearly overlapping, because group D is widespread.

The PCA created three sub-plots offering a low dimensional representation of all the samples. Denoting PC1, PC2, and PC3 as the first three principal components, Figure 6 shows the projection of the samples in the PC1 versus PC2 and PC1 versus PC3 spaces, respectively. From the figure, it is possible to notice a clear overlap of the groups A-B. Instead, groups C-D are not entirely overlapping because of the spread distribution of group D samples. For completeness, Supplementary Figure 3 illustrates the projection in the PC2 versus PC3 space.

**FIGURE 7:**
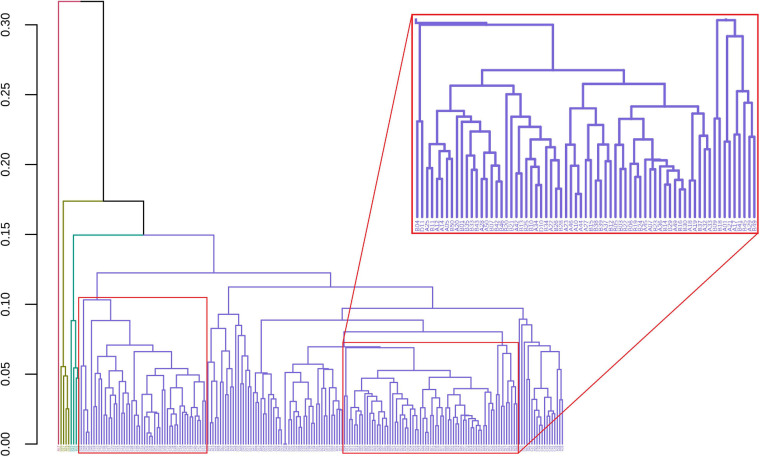
Samples dendrogram for Case Study 1 (ovarian cancer). On the left, the plot shows the overall dendrogram, where we enlighten two groups of samples in red rectangles. The red rectangle on the left is a cluster of samples from groups C to D. The red rectangle on the right (zoomed) represents a cluster of samples from groups A to B.

GeenaR clustered the samples assuming that the number of clusters is known and equal to four (*k* = 4). Figure 7 shows the final dendrogram, where we enlighten two red rectangles. The complete dendrogram on the left shows how the value of 4 clusters could be incorrect, because of a bunch of mass spectra that probably seems outliers. The zoom reported in Figure 7 depicts a cut in the dendrogram that can be considered a good cluster since it is mostly composed of samples from groups A-B (68 out of 100). Moreover, the red rectangle on the left depicted a higher cut (not reported as zoom) that consisted completely of samples from groups C-D (50 out of 100).

### Case Study 2: Colorectal Cancer

Glycans are polysaccharides conjugated with proteins, lipids, and proteoglycans. Their profile, in terms of expression, changes during the proliferation of cancer. Thus, they can be considered as biomarkers for the study of pathology evolution and the development of new treatments. We selected a study where researchers used mass spectrometry to analyze differences in N-glycan profiles between tumor and healthy samples. More details are reported in Holm et al. (2020).

The dataset consisted of 47 mass spectra divided into two different groups of different sizes: 37 with colorectal cancer and 10 healthy colon tissue samples (from patients analyzed in a previous workflow by the authors). Furthermore, 19 tumor samples were from the right-side colon and 17 tumor sample were from the left-side colon, with an additional difference in the stage of the tumor (19 for stage II and 17 for stage III); five healthy samples were from the right-side colon and five healthy samples were from the left-side colon. The mass spectra were generated by Matrix-assisted Laser Desorption and Ionization Time-Of-Flight (MALDI-TOF) mass spectroscopy technique, in order to show N-glycan profiles. Each mass spectrum consisted of around 175,000 values, in the range 500–5,000 m/z. In this example, we jointly analyzed with GeenaR the mass spectra derived from *ProteomeXchange*, *ProteomeCentral*, accession number PXD018673 (repository link: http://www.proteomexchange.org/, dataset link: http://proteomecentral.proteomexchange.org/cgi/GetDataset?ID=PXD018673).

After compiling a target file reporting the file names and the reference group for each mass spectrum, we executed GeenaR with the following choices:

A. we selected all the steps, skipping the average step;
B. we trimmed the raw mass spectra is in the range 500–3,500;
C. we applied the square root method for stabilization, Savitzky-Golay method with a half window of 10 points for smoothing, SNIP method with 25 iterations for baseline correction, TIC method for normalization;
D. we used MAD method with a half window size of 20 points, 2 as SNR, tolerance of 0.002 for the noise estimation, and lowess method for the phase correction, both in alignment step;
E. we chose the strict method for peak binning and 50% of coverage for peak selection;
F. we did not apply any clustering algorithm when plotting the heatmap;
G. we used the average method as the link function for clustering, with *k* = 2 as suggested using the silhouette statistic technique.

**FIGURE 8:**
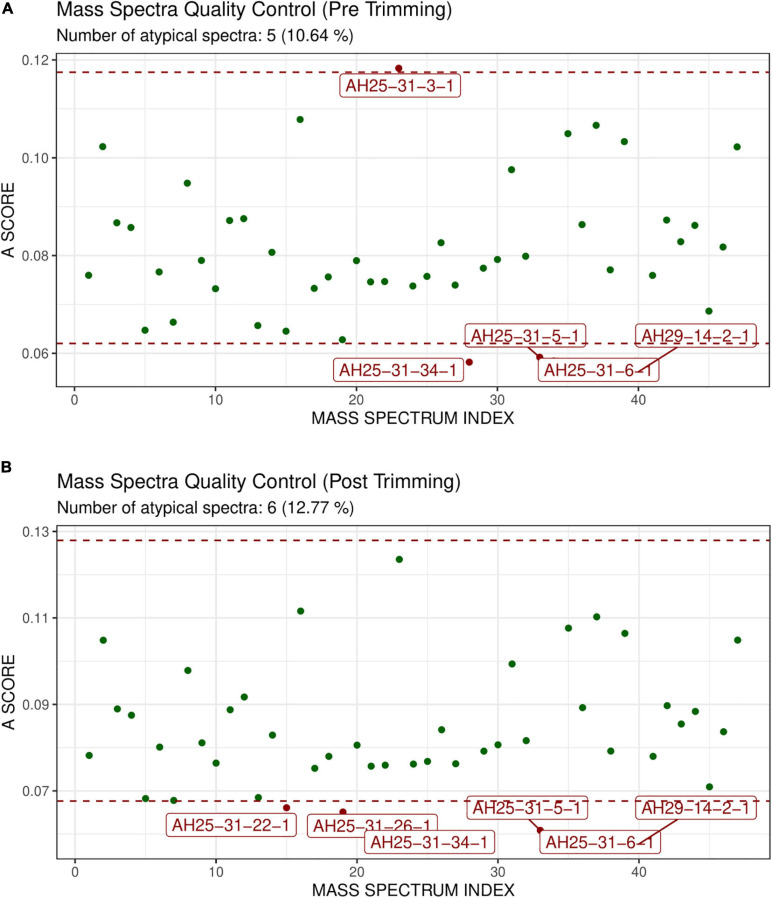
Atypicality score plot without trimming **(A)** and with trimming **(B)** for Case Study 2 (colorectal cancer). Similar to Figure 4. **(A)** The mass spectra signed AH25-31-1, AH25-31-34-1, AH25-31-5-1, AH25-31-6-1, AH29-14-2-1 are the possible outliers. **(B)** The m/z range of trimming is 500–3,500. The mass spectra signed as AH25-31-22-1, AH25-31-26-1, AH25-31-34-1, AH25-31-5-1, AH25-31-6-1, AH29-14-2-1 are the possible outliers.

GeenaR did not detect any empty mass spectra. The quality control pre-trimming identified as possible outliers samples AH25-31-3-1, AH25-31-34-1, AH25-31-5-1, AH25-31-6-1, AH29-14-2-1, enlighten in Figure 8A. The potential outliers represent about 11% of the mass spectra (5 out of 47). The quality control post-trimming, with trimming in the m/z range of 500–3,500, showed how cutting the noise toward the tail can modify the study, changing which mass spectrum can be tagged as potential outliers. In this case (see Figure 8B), samples AH25-31-22-1, AH25-31-26-1, AH25-31-34-1, AH25-31-5-1, AH25-31-6-1, AH29-14-2-1 are marked as potential outliers, but not removed from the rest of the analysis. They represent about 13% of the mass spectra (6 out of 47).

**FIGURE 9:**
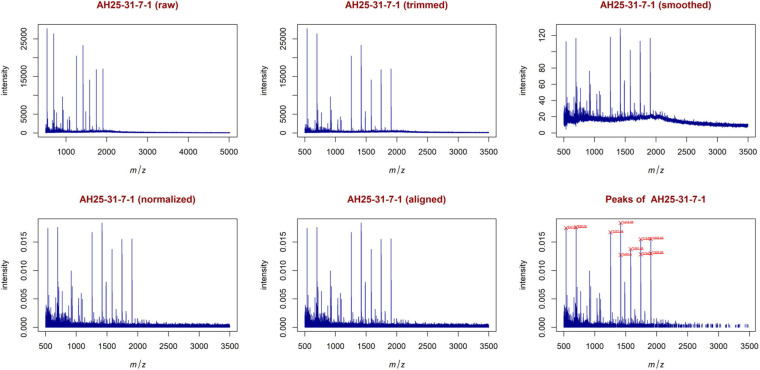
Examples of plots created during the entire analysis for Case Study 2 (colorectal cancer). Similar to Figure 5, with sample AH25-31-7-1. The most relevant peaks with the 10 highest peaks enlighten in red are: 537.05, 699.06, 1257.44, 1419.49, 1420.50, 1581.55, 1743.60, 1744.60, 1905.65, 1906.66.

In this case, the feature matrix consisted of 47 mass spectra and 1,779 relevant peaks, that represent all the analyzed mass spectra. Figure 9 shows the mass spectrum of sample AH25-31-7-1 (m/z versus intensity) as raw, trimmed, smoothed, normalized, and aligned, respectively. Moreover, it also shows the list of relevant peaks identified in the sample. The number of peaks is very high, but the highest peaks (signals) are recognizable.

From the feature matrix, GeenaR created the heatmap shown in Supplementary Figure 4 that allows identifying visually which peaks are essential for some mass spectra. Here, since the number of peaks in the feature matrix is high, the heatmap was transposed and depicted only the m/z range of 500–675 (the complete heatmap is available in Supplementary Figure 5). It is possible to notice that the most informative peaks are: 537.0479, 551.0338, 699.0588, 771.2873, 875.0307, 917.3404, 933.3372, 1037.0503, 1079.3900, 1095.3845, 1136.4096, 1175.3823, 1257.4398, 1419.4942, 1581.5488, 1647.5956, 1663.5933, 1743.5995, 1744.6009, 1809.6507, 1905.6517, 1906.6568. The peaks after the m/z value 2,000 can be considered as low signals.

**FIGURE 10:**
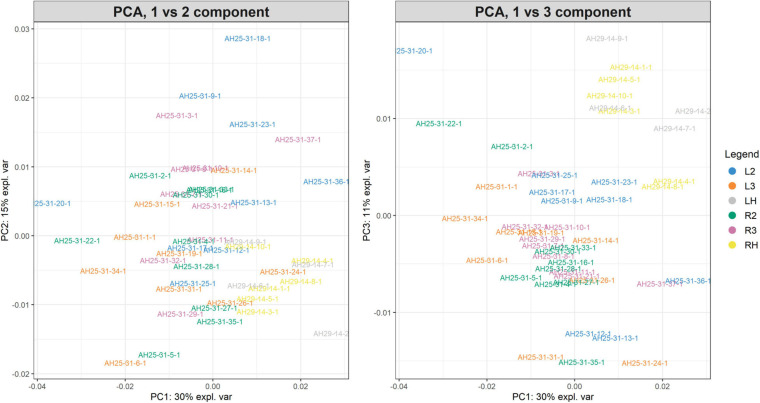
PC1vsPC2 and PC1vsPC3 plots for Case Study 2 (colorectal cancer). Similar to Figure 7. The PCA analysis of the feature matrix revealed that PC1, PC2, PC3 accounted for 56% of the total explained variance. Colorectal cancer groups (L2, L3, R2, R3) are less overlapping, whilst control groups (LH, RH) are overlapping in the IV quadrant (left panel) and in the I quadrant (right panel).

The PCA created three sub-plots offering a low dimensional representation of all the samples. Figure 10 shows the projection of the samples in the PC1 versus PC2 and PC1 versus PC3 spaces, respectively. As shown, it is possible to see a clear superimposition of LH-RH groups located in both panels. Supplementary Figure 6 shows the projection in the PC2 versus PC3 space.

**FIGURE 11:**
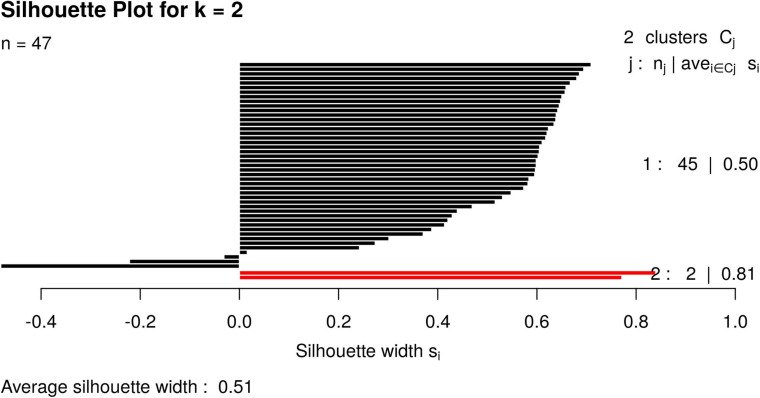
Silhouette plot for Case Study 2 (colorectal cancer). Silhouette plot with the estimate of the best number of clusters k with the silhouette method: in this case, k = 2.

**FIGURE 12:**
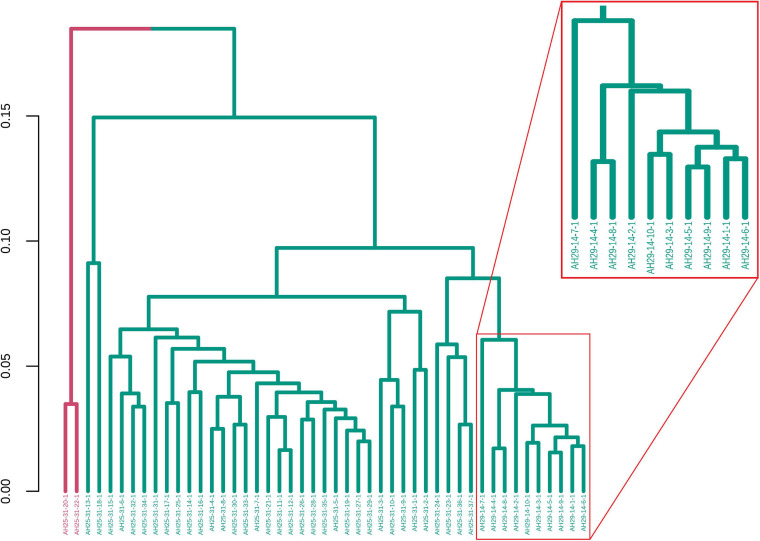
Samples dendrogram for Case Study 2 (colorectal cancer). On the left, the plot shows the overall dendrogram, where we enlighten one group of samples in a red rectangle. This group consists of the cluster with samples from groups LH-RH. The other groups are mixed.

For illustrative purposes, we assume that the number of groups was unknown. Therefore, GeenaR evaluated the silhouette statistics for a range of possible values of k and showed the silhouette plot for the best value of k. Figure 11 shows the silhouette plot for *k* = 2. Using this value as the number of clusters, Figure 12 shows the final dendrogram, where we enlighten one red rectangle. The zoom reported in the dendrogram inside the red rectangle depicts a cut that can be considered a perfect cluster since it is composed of samples from groups LH-RH (10 out of 10).

### Reporting

As already mentioned, GeenaR produces analysis reports in .html file format that allows the user to keep track of the steps and parameters used during the analysis, and to fully reproduce the results from the version with the R code. The report is created by agglomerating all the results and plots obtained with GeenaR. The report relative to case study 2 (colorectal cancer) is available in Supplementary Report. As shown, the table of content of the report is composed of 10 paragraphs: (1) main loaded packages, (2) selected tasks and used parameters, (3) acquisition of the mass spectra, (4) quality control on mass spectra and possible outliers, (5) all the processes to clean the mass spectra, (6) all the processes to average/align the mass spectra, (7) peak detection, (8) unsupervised analysis and clustering, (9) links to download the log file, the feature matrix and all the mass spectra (raw and cleaned), (10) session information about R environment. For completeness, we remember that the version of the report with the code has not the embedded plots of the mass spectra (available in the version of the report without the code).

### Performance Assessment

To assess the performances of GeenaR, we performed two tests. In the first, we executed three times the analysis of each case study. We computed the average execution times and comparatively analyzed the time required for the individual analysis steps. In the second, we created various subsets of different sizes of an existing large spectra dataset related to a study on colorectal cancer (Beitia et al., 2020). We investigated how the execution times vary according to the dimension of the subset. We carried out the full analysis in both tests, meaning that we required the system to perform all analysis steps, including reading mass spectra, quality control, trimming, cleaning, aligning, peak extraction, clustering, and report.

**FIGURE 13:**
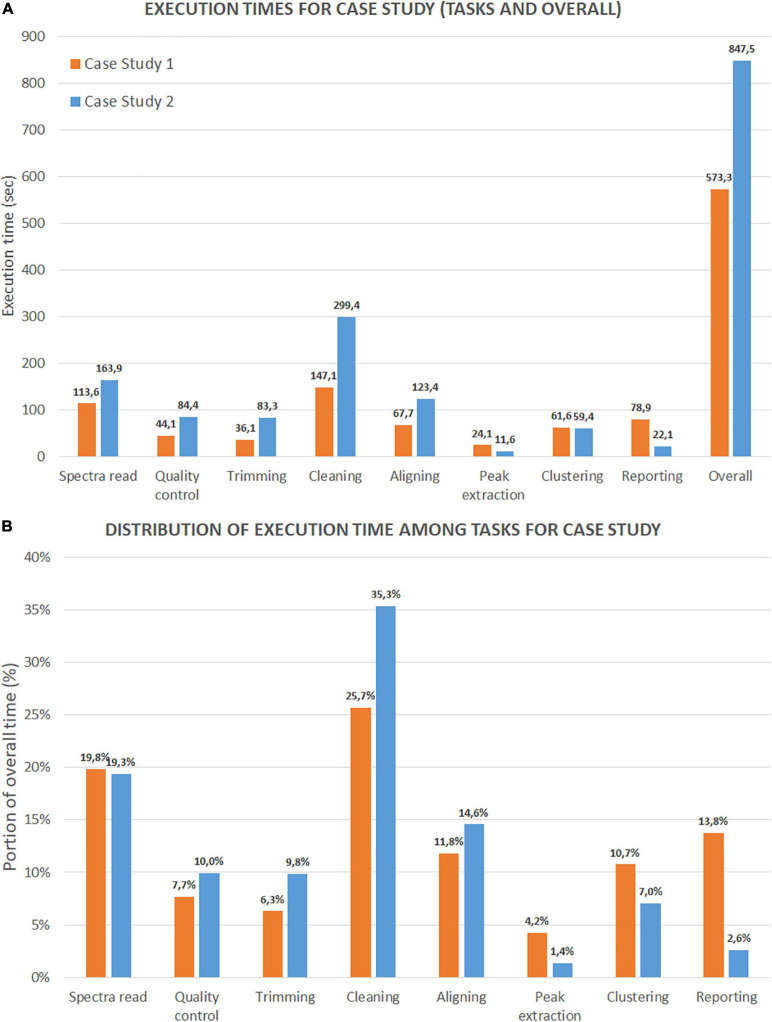
Time performance comparison between Case Study 1 (ovarian cancer) and Case Study 2 (colorectal cancer). **(A)** Shows the execution time in seconds for each step and for the entire process. **(B)** Shows the portion of execution time in percentage compared to the overall execution time.

We present the first test results on execution time in Figure 13, with two sets of histograms. Figure 13A reports the execution times (in seconds) of each analysis step and the overall time (last column). The three most demanding steps are cleaning (including variance stabilization, smoothing, baseline correction, and normalization), spectra reading, and aligning (without the averaging task). It is noteworthy that case study 2 shows a slower overall execution despite a lower number of mass spectra (47 spectra versus 200 for case 1). The reason is due to the dimension of the file for each mass spectrum (which is much larger). Figure 13B reports the distribution of execution times for each step as a percentage of the overall execution time. For case study 1, cleaning, spectra reading, and aligning steps account for 25.7, 19.8, and 11,8% of the execution time, respectively. For case study 2, the same steps account for 35.3, 19.3, and 14.6%. As expected, the number of mass spectra has a notable impact on the reporting percentage for case study 1. This result is because the report with mass spectra embeds all the plots from raw mass spectra to peak lists.

**FIGURE 14:**
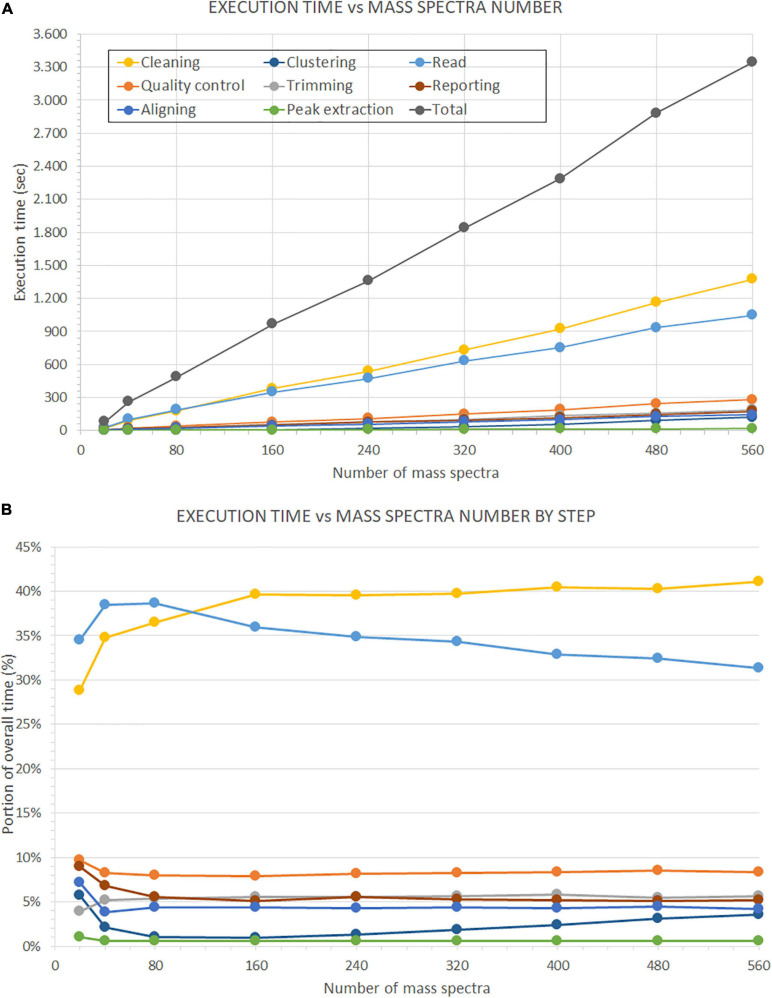
Time performance comparison on the number of processed mass spectra. This evaluation is performed on the dataset described in Beitia et al. (2020). **(A)** Shows the performance of GeenaR to changes in the number of processed mass spectra. **(B)** Shows the impact of each step in percentage compared to the overall execution time. The legend of the colors is reported in Panel A.

Moreover, we present the second test results on the number of mass spectra in Figure 14. In the upper plot (Figure 14A), we show the evolution of the overall execution time and the execution time of each analysis step, with the increase of the number of mass spectra. We evaluated seven subsets of samples (i.e., 5, 10, 20, 40, 60, 80, 100, 120, and 140). Each sample consists of four replicates. All times increase in a linear progression. Indeed, we observed a correlation factor of 0.999 between the number of mass spectra and the overall execution time, and correlation factors from 0.943 to 1.000 between the number of mass spectra and the single analysis steps.

Furthermore, linear regression analysis showed different speed increases for the steps leading, e.g., the time requested for clustering data overcame the time for reading data for a number of mass spectra greater than about 160 (data not shown). Figure 14B shows the execution time of each step as a percentage of the overall execution time. The time associated with the cleaning and reading steps has the highest impact on the overall execution time. The most important evidence is the inversion of the trends for the previous steps at around 120 mass spectra: after this value of the number of mass spectra, the cleaning step has a 40% impact on the overall execution time, while the reading step tends to decrease progressively. Times associated with all other steps remain almost stationary within the range 0–9% of the overall execution time, independently from the number of mass spectra: only the clustering step seems to show a slight increasing slope.

## Discussion

Mass spectrometry is an analytic technique used in many biological fields, which produces a massive quantity of data with a particular connection with proteomic data. MALDI-TOF is one the most used combined technology in mass spectrometry, with many advantages in obtaining results in a short time, with high resolution, and good accuracy. The main results from the spectrometer are raw mass spectra, represented by a list of intensities for different mass-to-charge ratio (m/z) values. The analysis of raw mass spectra requires the application of several computational methods to correct or reduce different kinds of noise, which can affect data. Many software, open-source or not, are available for the treatment of the raw mass spectra, such as the abovementioned *MaxQuant* and *OpenMS*, which can be considered the gold standard in pre-processing, visualizing, and analyzing different kinds of mass spectra. Nevertheless, although these tools incorporate a great number of algorithms and functionalities, they are not straightforward to use, and scientists need a significant effort in studying manuals or attending courses before using available tools for the analysis of their data. Moreover, they also require to set-up a specific computational environment for their usage (from their installation to the computational resources for their execution) which might constitute another limit.

Geena2 is a straightforward tool for analyzing the MALDI-TOF mass spectra, a revised, more efficient, and user-friendly version of Geena. It is available as an open-access web-server application, hence its usage does not require any installation or computational resource from the user. Its main output consists in the identification of peaks common among the mass spectra so that a differential analysis can be carried out between groups of spectra. The robust architecture of Geena2, both in terms of the web platform and the background layer structure, suggested us to implement new functionalities, with a better focus on the visualization of mass spectra, the statistical analysis, and the reproducibility of the findings. GeenaR is our new tool that copes with all these features. It combines the architecture in Geena2 with the power of the R environment. The strong points of GeenaR are:

1. *User-friendliness*. GeenaR follows the user during the entire process, from the upload of the raw mass spectra to the visualization of results. Thanks to a simple user-friendly web page, the workflow is explained in all its features, placing particular importance on the format of the files to upload (mass spectra, target file, and attributes file) and on the selection of steps, methods and parameters.
2. *Multi-methods.* The potentialities of R language and packages allowed us to provide different methods to pre-process and analyze mass spectra, and visualize results. All the methods are immediately available and selectable on the web page. Two case studies illustrate some of the different functionalities that are available.
3. *Modularity*. The structure of GeenaR is modular: each module performs one or more related tasks. The intermediate output of each task is saved in a devoted folder and transferred to the following module. This architecture allows us to add new modules anytime, implementing the corresponding R functions and revising the interface accordingly.
4. *Hardware requirements*. GeenaR is on a host server, thus the user does not need to download and install any tool, as it happens for many software available online. Moreover, the user has no constraints on the local hardware to run the software: a standard browser is sufficient for a run of GeenaR, obtaining all the results in .html format (readable on the browser itself);
5. *Computational reproducibility.* GeenaR supports the Reproducible (computational) Research improving transparency, knowledge transfer, and reproducibility of findings. For each job, GeenaR produces a human-readable report that embeds the results with the selection of parameters. Note that there is also a version of the analysis report that includes the R code used to process the mass spectra. The user can also re-execute the code on a local machine with minimal experience with the R language, such as suggested in Del Prete et al. (2018). Moreover, researchers can use the report as supplementary material in publications such as we did in the context of RNA-seq data analysis (Costa et al., 2017). The reproducibility of the computational analysis constitutes one of the main advantages of GeenaR.

We demonstrated with the proposed two oncological case studies that GeenaR can handle different resolutions in mass spectra. Our results showed how it is possible to determine outliers, visualize all the profiles (tumor and healthy samples), make available a series of unsupervised techniques such as PCA, heatmaps and cluster the mass spectra by their fingerprints obtained from the feature (peak) matrix.

Finally, we are aware that several improvements are possible in GeenaR. For example, we plan to include some supervised analysis methods as a novel module inside our pipeline, add different techniques for the selection of the most relevant peaks and for the computation of the similarity matrix. Moreover, although GeenaR is efficient, we plan to parallelize some parts of the work to reduce the bottlenecks: importing the mass spectra in *MassSpectrum* class and coping with the dimension of the report embedded with files of all the mass spectra. For this version of the tool, we set to 512 MB the maximum size of the compressed archive of the mass spectra to be uploaded for the analysis. However, the performances of the tool can be limited by various parameters, including the number of mass spectra of the dataset under analysis, the resolution for each mass spectrum, and the number of signals detected as relevant for the clustering analysis, which make up the feature matrix. For this reason, we could not yet define the exact limitations of the system. Nevertheless, we stressed the analysis up to 560 mass spectra without issues. We plan to further investigate on the limitations of the system and eventually make upgrades so that the number of mass spectra under analysis can be safely increased. Nevertheless, we firmly believe that GeenaR can help scientists to analyze proteomic datasets in a reproducible and simple way.

## Data Availability Statement

Publicly available datasets were analyzed in this study. This data can be found here: CLINICAL PROTEOMICS PROGRAM DATABANK–PROTEOMIC PATTERNS, https://home.ccr.cancer.gov/ncifdaproteomics/OvarianDataset4-3-02.zip and PROTEOMEXCHANGE–PROTEOME CENTRAL, http:// proteomecentral.proteomexchange.org/cgi/GetDataset?ID=PXD 018673.

## Author Contributions

EDP designed the study and implemented the workflow, performed analysis of the case studies, selected and discussed results, and wrote the manuscript. AF motivated and designed the study, contributed to the design of the workflow, the analysis of the case studies, the selection and discussion of results, and wrote the manuscript. AP motivated the study and contributed to the selection and discussion of the case studies. CA designed the study, contributed to the design of the workflow, co-supervised the implementation, selection and discussion of results, and wrote the manuscript. PR motivated and designed the study, contributed to the design of the workflow, implemented the user interface, co-supervised the implementation, selection, and discussion of results, and wrote the manuscript. All authors contributed to the article and approved the submitted version.

## Conflict of Interest

The authors declare that the research was conducted in the absence of any commercial or financial relationships that could be construed as a potential conflict of interest.
