# Supplementary material for: GeenaR: A Web Tool for Reproducible MALDI-TOF Analysis
Source: Front Genet. 2021 Mar 29;12:635814. doi: 10.3389/fgene.2021.635814 (PMC8039533; doi:10.3389/fgene.2021.635814)
Supplement: Supplementary file 1 [file Data_Sheet_1.docx]

***Supplementary Material: Supplementary Figures***


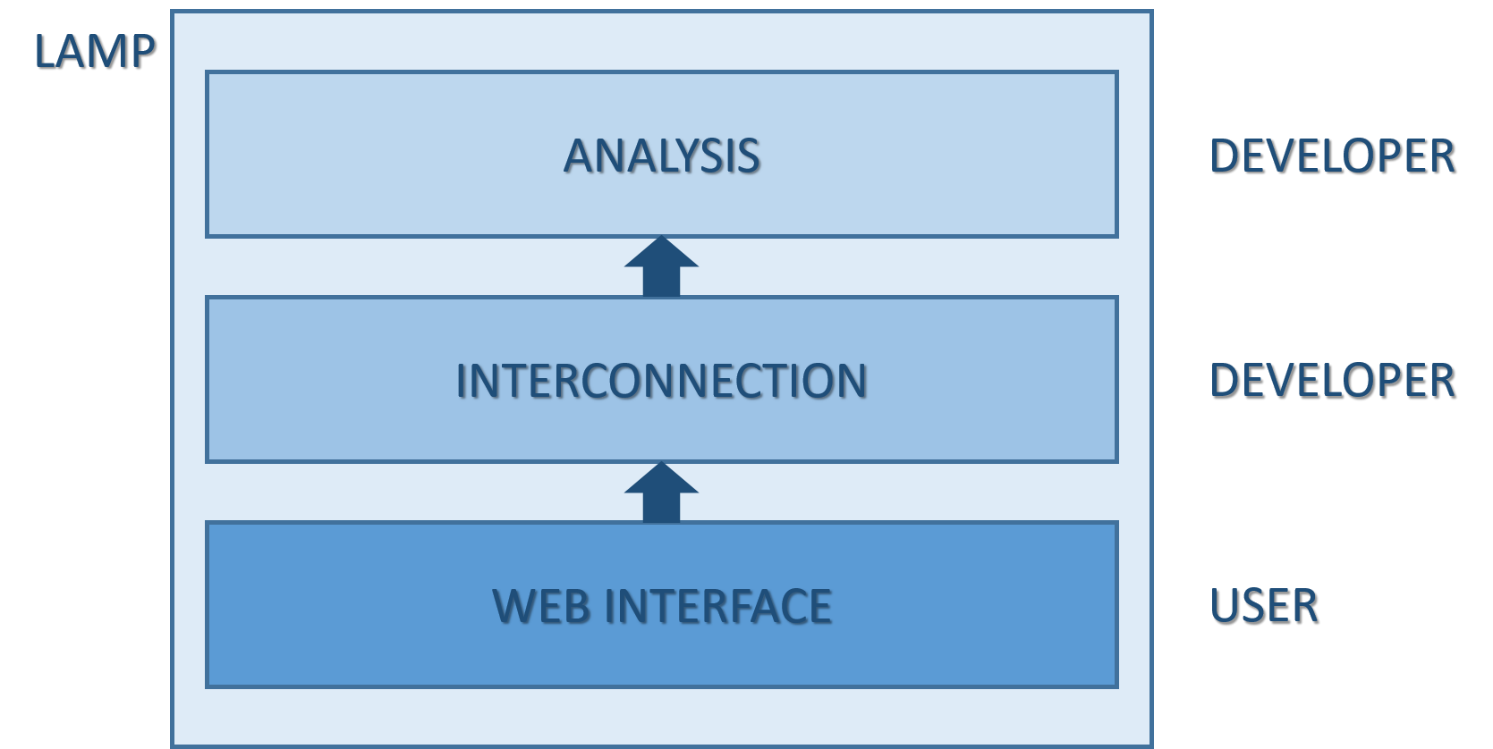


**Supplementary Fig.1. Overall structure of GeenaR.** Three different layers are inside the LAMP box. The Web Interface layer is linked to user’s choice of the methods and parameters; the Analysis and the Interconnection layers are not visible to the user and are linked to the connection step of the user’s choice, the pre-processing and analysis of the MALDI-TOF mass spectra, and the storage of the results.


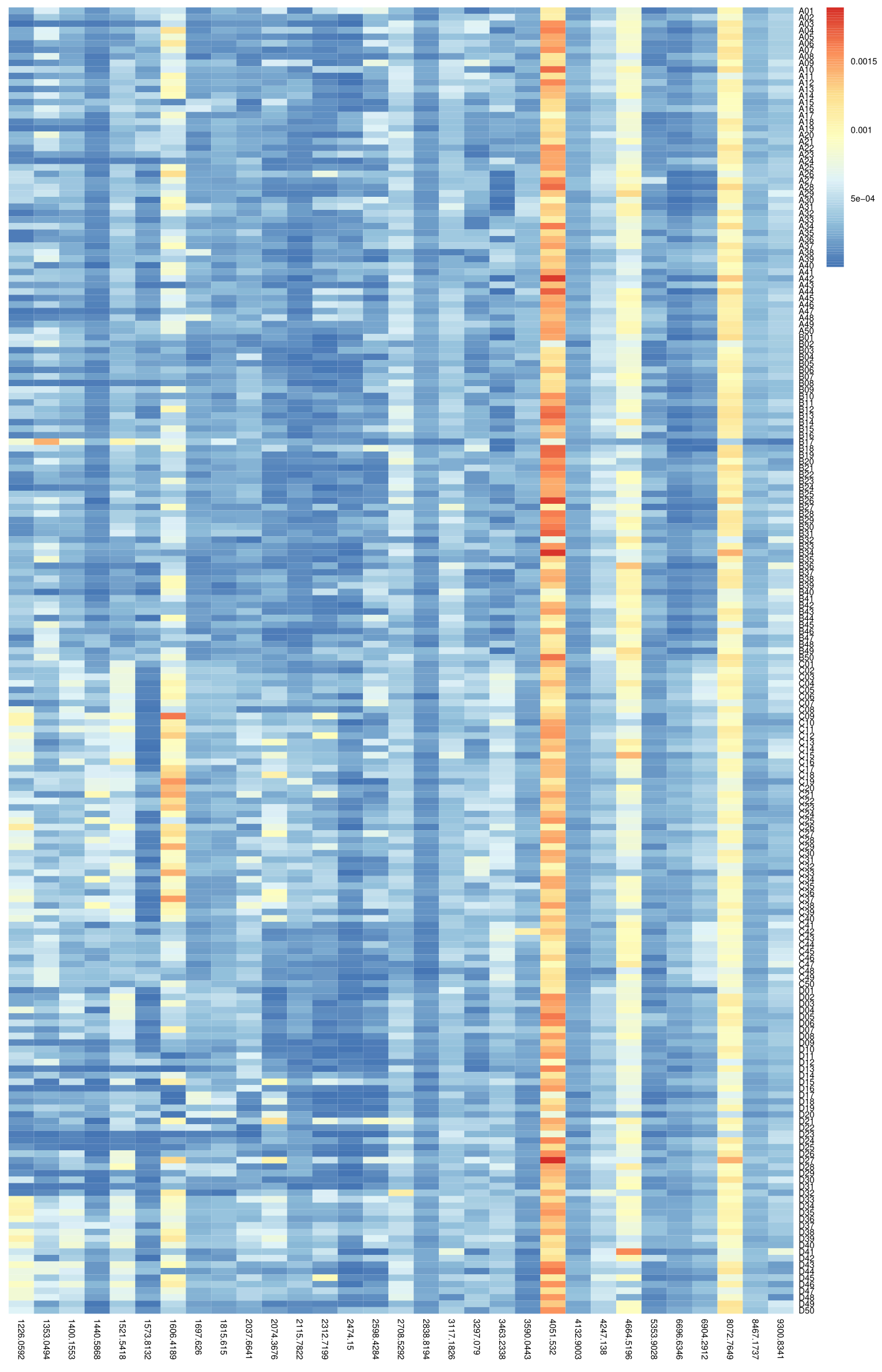


**Supplementary Fig.2. Peak intensity heatmap of the feature matrix for Case Study 1 (ovarian cancer).** Rows represent the samples, columns represent the relevant peaks in the feature matrix. Colors represent the normalized intensity.


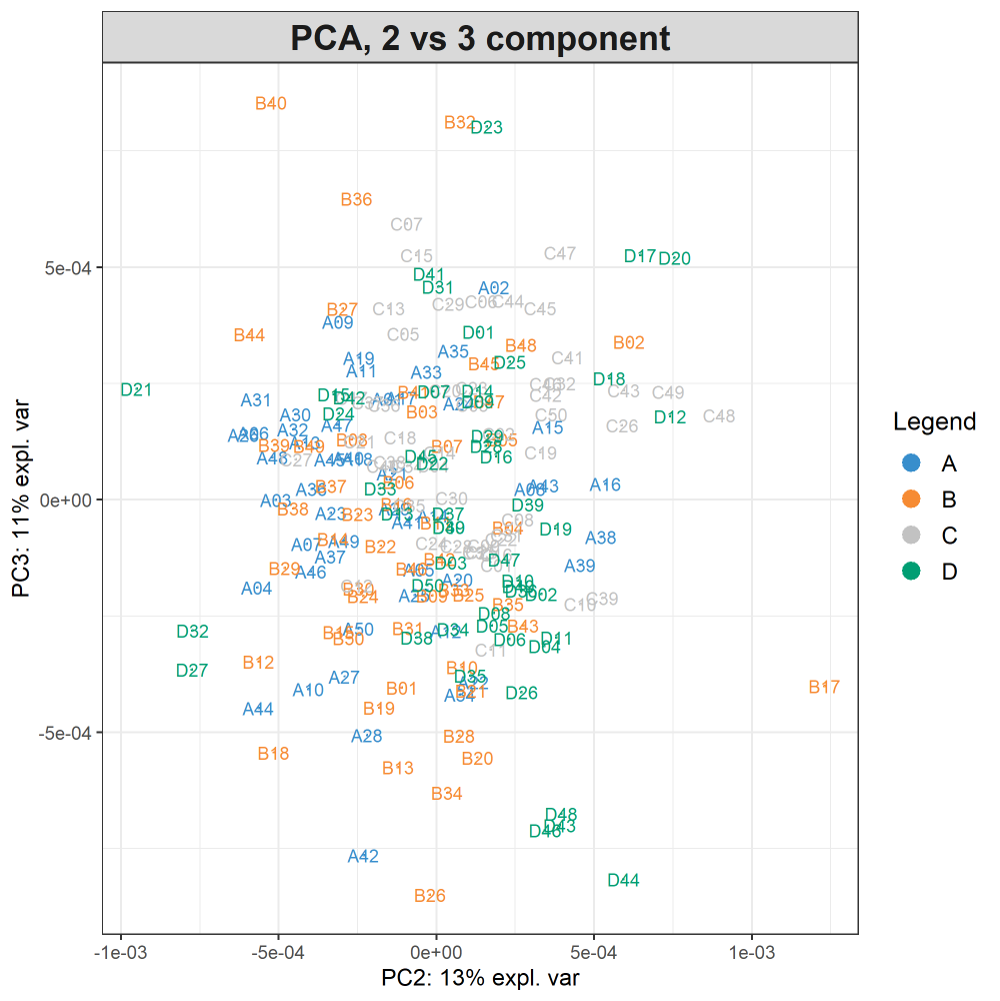


**Supplementary Fig.3. PC2vsPC3 plot (ovarian cancer).** The total explained variance for PC2 and PC3 is 24%. The legend on the right shows the four groups in terms of colors. Ovarian cancer groups (A, B) and control groups (C, D) are superimposed.


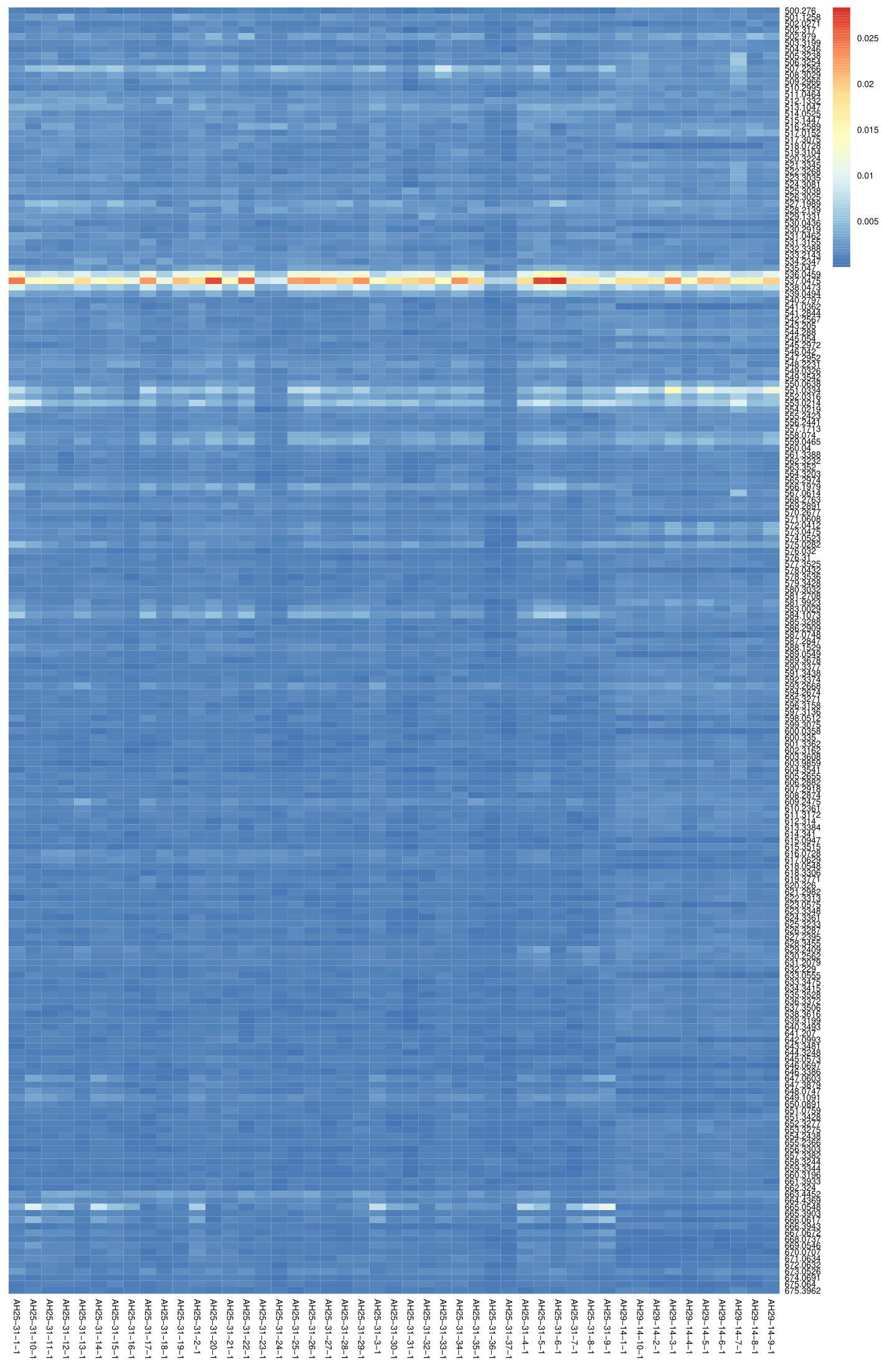


**Supplementary Fig.4. A subset of the peak intensity heatmap of the feature matrix Case Study 2 (colorectal cancer).** Similar to Supplementary Fig.2. The heatmap contains only relevant peaks in the 500-675 m/z range.


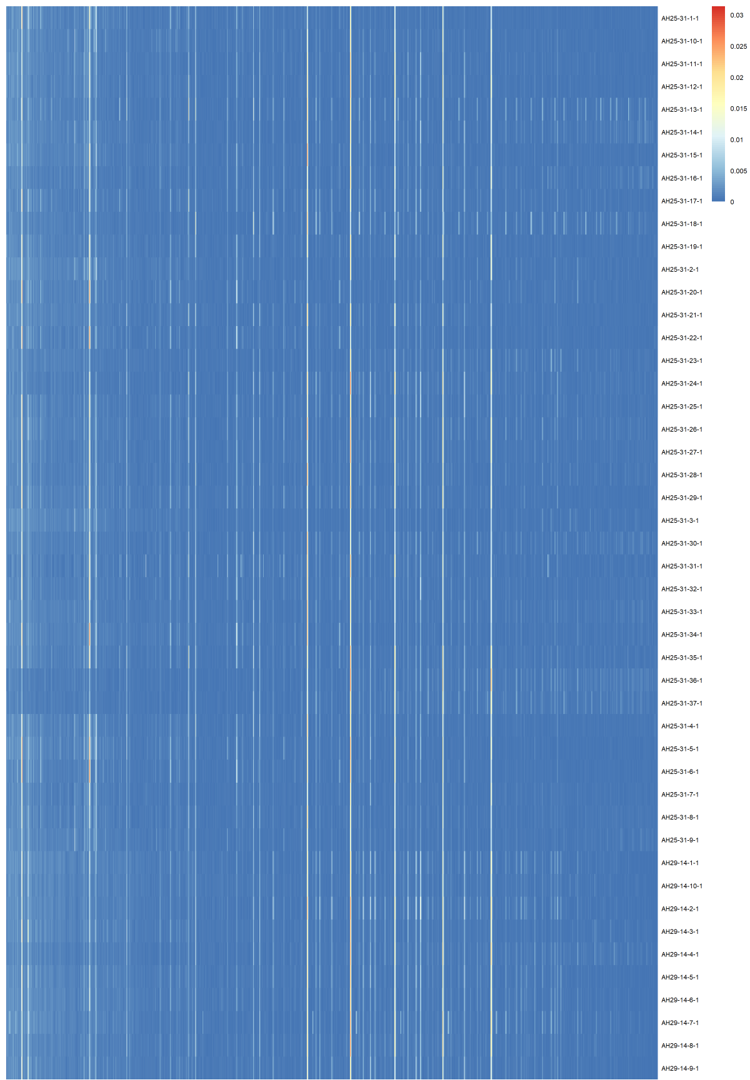


**Supplementary Fig.5. Complete peak intensity heatmap of the feature matrix Case Study 2 (colon-rectal cancer).** Similar to Supplementary Fig.2.


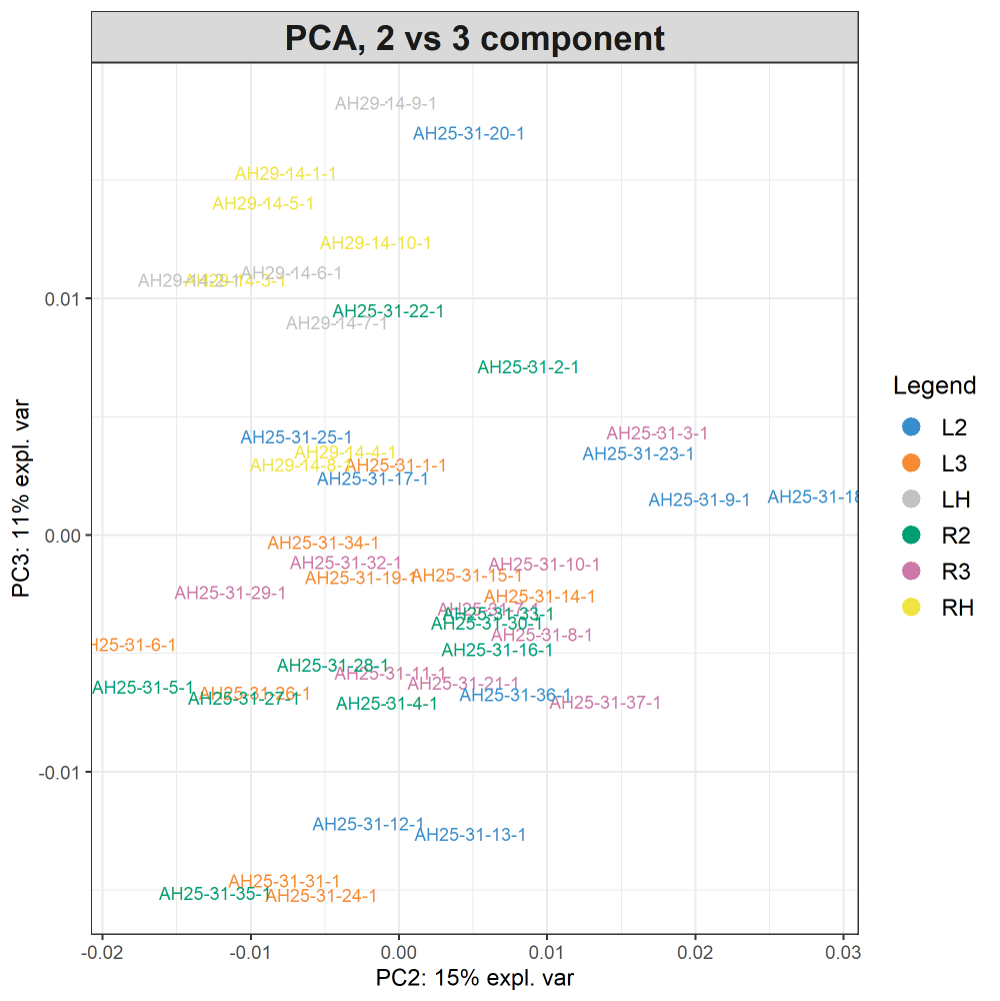


**Supplementary Fig.6. PC2vsPC3 plot (colon-rectal cancer).** The total explained variance for PC2 and PC3 is 26%. The legend on the right shows the four groups in terms of colors. Colon-rectal cancer groups (L2, L3, R2, R3) are less superimposed, whilst control groups (LH, RH) are superimposed in the II quadrant.
