# Supplementary material for: GeenaR: A Web Tool for Reproducible MALDI-TOF Analysis
Source: Front Genet. 2021 Mar 29;12:635814. doi: 10.3389/fgene.2021.635814 (PMC8039533; doi:10.3389/fgene.2021.635814)
Supplement: Supplementary file 2 [file Data_Sheet_2.ZIP › geenar_report_html.html]

 

 

 

 
 
 
 


 GeenaR Report of the Process - Mass Spectra Version 

 
 
 
 
 
 
 
 
 
 

 
 
 


 


 


 

 

 


 

 


 


 


 GeenaR Report of the Process - Mass Spectra Version 
  Generated with all the mass spectra and the results about the requested analysis.  

 

 
 
  1) PACKAGES  
  2) TASKS AND PARAMETERS  
  3) MASS SPECTRA ACQUISITION  
  4) QUALITY CONTROL  
  5) CLEANING MASS SPECTRA  
  6) AVERAGING AND ALIGNMENT  
  7) PEAK DETECTION  
  8) DATA EXPLORATION AND CLUSTERING  
  9) DOWNLOADING LOG CONTROL FILE, FEATURE MATRIX AND MASS SPECTRA PLOTS  
  10) SESSION INFO  
 
 

 
 1) PACKAGES 
 The main packages used for the analysis are: 
 
 MALDI-TOF mass spectra treatment ->  MALDIquant ,  MALDIquantForeign ,  MALDIrppa  
 similarity matrix generation ->  lsa  
 clustering ->  cluster ,  pheatmap ,  dendextend  
 general plotting ->  ggplot2 ,  ggrepel  
 data exploration ->  mixOmics  
 table generation ->  knitr ,  kableExtra  
 
  library(MALDIquant)
library(MALDIquantForeign)
library(MALDIrppa)
library(cluster)
library(scales)
library(ggplot2)
library(ggrepel)
library(dendextend)
library(mixOmics)
library(lsa)
library(knitr)
library(kableExtra)  
 
 
 2) TASKS AND PARAMETERS 
 Briefly, the main tasks are reported in the first column (TASK NAME) and the second column (CHOICE) shows if the tasks are selected or not by the user. The third column (METHOD I) and the fourth column (METHOD II) show the methods for the related task. Numeric parameters are collected from the fifth column to the seventh column (PARAMETER I,II,III). Following, the list of tasks, methods and parameters set by the user are: 
 
 
 
 
Task
 
 
Choice
 
 
Method I
 
 
Method II
 
 
Parameter I
 
 
Parameter II
 
 
Parameter III
 
 
 
 
 
 
quality_pre
 
 
yes
 
 
 
 
 
 
 
 
 
 
 
 
 
 
trimming
 
 
yes
 
 
 
 
 
 
500
 
 
3500
 
 
 
 
 
 
quality_post
 
 
yes
 
 
 
 
 
 
 
 
 
 
 
 
 
 
stabilization
 
 
yes
 
 
sqrt
 
 
 
 
 
 
 
 
 
 
 
 
smoothing
 
 
yes
 
 
SavitzkyGolay
 
 
 
 
10
 
 
 
 
 
 
 
 
baseline
 
 
yes
 
 
SNIP
 
 
 
 
25
 
 
75
 
 
 
 
 
 
normalization
 
 
yes
 
 
TIC
 
 
 
 
 
 
 
 
 
 
 
 
average
 
 
no
 
 
median
 
 
 
 
 
 
 
 
 
 
 
 
alignment
 
 
yes
 
 
MAD
 
 
lowess
 
 
20
 
 
2.0
 
 
0.002
 
 
 
 
peak
 
 
yes
 
 
strict
 
 
 
 
0.5
 
 
 
 
 
 
 
 
pca
 
 
yes
 
 
 
 
 
 
 
 
 
 
 
 
 
 
clustering
 
 
yes
 
 
average
 
 
sil
 
 
4
 
 
 
 
 
 
 
 
heatmap
 
 
yes
 
 
 
 
 
 
0
 
 
 
 
 
 
 
 
reporting
 
 
yes
 
 
yes
 
 
 
 
 
 
 
 
 
 
 
 
 
 
 3) MASS SPECTRA ACQUISITION 
 The MALDI-TOF mass spectra uploaded by the user are 47: 
  ##  [1] "AH25-31-1-1.mzXML"  "AH25-31-10-1.mzXML" "AH25-31-11-1.mzXML"
##  [4] "AH25-31-12-1.mzXML" "AH25-31-13-1.mzXML" "AH25-31-14-1.mzXML"
##  [7] "AH25-31-15-1.mzXML" "AH25-31-16-1.mzXML" "AH25-31-17-1.mzXML"
## [10] "AH25-31-18-1.mzXML" "AH25-31-19-1.mzXML" "AH25-31-2-1.mzXML" 
## [13] "AH25-31-20-1.mzXML" "AH25-31-21-1.mzXML" "AH25-31-22-1.mzXML"
## [16] "AH25-31-23-1.mzXML" "AH25-31-24-1.mzXML" "AH25-31-25-1.mzXML"
## [19] "AH25-31-26-1.mzXML" "AH25-31-27-1.mzXML" "AH25-31-28-1.mzXML"
## [22] "AH25-31-29-1.mzXML" "AH25-31-3-1.mzXML"  "AH25-31-30-1.mzXML"
## [25] "AH25-31-31-1.mzXML" "AH25-31-32-1.mzXML" "AH25-31-33-1.mzXML"
## [28] "AH25-31-34-1.mzXML" "AH25-31-35-1.mzXML" "AH25-31-36-1.mzXML"
## [31] "AH25-31-37-1.mzXML" "AH25-31-4-1.mzXML"  "AH25-31-5-1.mzXML" 
## [34] "AH25-31-6-1.mzXML"  "AH25-31-7-1.mzXML"  "AH25-31-8-1.mzXML" 
## [37] "AH25-31-9-1.mzXML"  "AH29-14-1-1.mzXML"  "AH29-14-10-1.mzXML"
## [40] "AH29-14-2-1.mzXML"  "AH29-14-3-1.mzXML"  "AH29-14-4-1.mzXML" 
## [43] "AH29-14-5-1.mzXML"  "AH29-14-6-1.mzXML"  "AH29-14-7-1.mzXML" 
## [46] "AH29-14-8-1.mzXML"  "AH29-14-9-1.mzXML"  
 All the mass spectra have been converted in the S4 class type ‘MassSpectrum’, with the information for each mass spectrum about: 
 
 number of the m/z values 
 range of the m/z values 
 range of the intensity values 
 dimension of the file. 
 
 The information of the first mass spectrum is reported for showing the ’MassSpectrum" class type: 
  ## S4 class type            : MassSpectrum      
## Number of m/z values     : 176185            
## Range of m/z values      : 499.971 - 5018.735
## Range of intensity values: 1e+00 - 5.308e+04 
## Memory usage             : 2.701 MiB  
 
 
 4) QUALITY CONTROL 
 The quality control on the uploaded mass spectra can be performed before and after the trimming task, in order to control if the mass spectra trimming affects the number of mass spectra that can be outliers for the dataset. 
 
 4.1) QUALITY CONTROL PRE-TRIMMING 
 The numbers of m/z values is shown per mass spectrum. Moreover, a control on empty mass spectra and on frequency of m/z values per mass spectrum (over a certain threshold) is reported. We suggest to remove empty mass spectra from the analysis; irregularities in frequency do not affect the following analysis. 
  ## 
## 161255 163239 165296 165500 168481 169394 171808 173705 174391 175338 
##      1      1      1      1      1      1      1      1      1      1 
## 175507 175931 175987 176020 176104 176118 176125 176134 176145 176148 
##      1      1      1      1      1      1      1      1      1      2 
## 176153 176159 176161 176163 176166 176169 176173 176174 176179 176181 
##      1      1      1      1      1      1      1      1      3      1 
## 176182 176185 176186 
##      1      7      6  
 Any empty mass spectrum? FALSE 
 Are all the mass spectra regular? FALSE 
 The atypicality score A, calculated by using the Rousseeuw’s Q robust scale estimator, is used in order to provide a range of acceptance for the mass spectra (between the dotted red lines). Only the outliers are depicted with the codename of the mass spectrum.  
 
 
 4.2) QUALITY CONTROL POST-TRIMMING 
 The trimming task is considered done even if the user does not provide a specific range of trimming (the mass spectra are considered in their entirety.) Nevertheless, the quality control after the trimming is still subject to the user’s choice. In this case the mass spectra are trimmed. 
 The atypicality score A is calculated after the trimming task. We suggest to compare the possible outliers between the two cases (before and after trimming), in order to decide if the related mass spectra should be eliminated from the study or not.  
 
 
 
 5) CLEANING MASS SPECTRA 
 It is possible to select four different tasks for cleaning the mass spectra. Briefly, the choice of the user is: 
 
 variance stabilization: YES; 
 smoothing: YES; 
 baseline correction: YES; 
 normalization: YES. 
 
 
 
 6) AVERAGING AND ALIGNMENT 
 The averaging task is based on the presence of different replicas for a single sample, and the user can put together this replicas in order to have one single averaged sample for each of them. In this case the mass spectra are NOT averaged on replicas for single sample. 
 The alignment task is performed among all the mass spectra, and it is compulsory to have a better detection of the most important peaks. 
 
 
 7) PEAK DETECTION 
 From the extraction of the most important peaks, a feature matrix is created, with the aligned mass spectra as rows and peaks as columns. The raw feature matrix is sparse: lacking values are integrated with a linear interpolation among the other peaks, in order to have a full matrix. 
 A quality pattern for the peak distribution over all the mass spectra is reported by a heatmap:  
 
 
 8) DATA EXPLORATION AND CLUSTERING 
 Principal Component Analysis is performed on mass spectra based on peaks information. The first three principal components are compared to each other.   Furthermore, the most important features for the principal components are depicted in the following loadings plots.    
 The following plot represents the number of clusters suggested by the method of k estimation selected by the user (reported in the title):  
 A dendrogram for the samples clustering is created by using the cosine correlation as similarity measure between two samples (peak list), and the clusters are coloured taking into account k (estimated or selected).  
 
 
 9) DOWNLOADING LOG CONTROL FILE, FEATURE MATRIX AND MASS SPECTRA PLOTS 
 The log control file, the feature matrix and all the mass spectra plots, from raw mass spectra to peak files (one zip file for each available type of processing, depending on the selected tasks), are available below for the downloading: 
  Download Log Control file    
  Download Feature Matrix file    
  Download Raw files    
  Download Trimmed files    
  Download Stabilized files    
  Download Smoothed files    
  Download Corrected files    
  Download Normalized files    
 No avereged mass spectra (no replicas)   
  Download Aligned files    
  Download Peak files    
 
 
 10) SESSION INFO 
 The information about OS, R version and loaded R packages is following reported: 
  ## R version 3.6.3 (2020-02-29)
## Platform: x86_64-pc-linux-gnu (64-bit)
## Running under: Ubuntu 18.04.5 LTS
## 
## Matrix products: default
## BLAS:   /usr/lib/x86_64-linux-gnu/blas/libblas.so.3.7.1
## LAPACK: /usr/lib/x86_64-linux-gnu/lapack/liblapack.so.3.7.1
## 
## locale:
##  [1] LC_CTYPE=en_US.UTF-8       LC_NUMERIC=C              
##  [3] LC_TIME=en_US.UTF-8        LC_COLLATE=en_US.UTF-8    
##  [5] LC_MONETARY=en_US.UTF-8    LC_MESSAGES=en_US.UTF-8   
##  [7] LC_PAPER=en_US.UTF-8       LC_NAME=C                 
##  [9] LC_ADDRESS=C               LC_TELEPHONE=C            
## [11] LC_MEASUREMENT=en_US.UTF-8 LC_IDENTIFICATION=C       
## 
## attached base packages:
## [1] stats     graphics  grDevices utils     datasets  methods   base     
## 
## other attached packages:
##  [1] knitr_1.23             kableExtra_1.1.0       pheatmap_1.0.12       
##  [4] lsa_0.73.2             SnowballC_0.7.0        mixOmics_6.8.5        
##  [7] MASS_7.3-51.4          dendextend_1.12.0      ggrepel_0.8.1         
## [10] ggplot2_3.2.0          scales_1.0.0           cluster_2.1.0         
## [13] MALDIrppa_1.0.1-2      lattice_0.20-38        wmtsa_2.0-3           
## [16] robustbase_0.93-5      signal_0.7-6           MALDIquantForeign_0.12
## [19] MALDIquant_1.19.3     
## 
## loaded via a namespace (and not attached):
##  [1] viridis_0.5.1            httr_1.4.0              
##  [3] tidyr_0.8.3              viridisLite_0.3.0       
##  [5] ellipse_0.4.1            assertthat_0.2.1        
##  [7] highr_0.8                yaml_2.2.0              
##  [9] pillar_1.4.2             backports_1.1.4         
## [11] glue_1.3.1               digest_0.6.20           
## [13] RColorBrewer_1.1-2       rvest_0.3.5             
## [15] colorspace_1.4-1         htmltools_0.3.6         
## [17] Matrix_1.2-17            plyr_1.8.4              
## [19] XML_3.98-1.20            pkgconfig_2.0.2         
## [21] purrr_0.3.2              corpcor_1.6.9           
## [23] webshot_0.5.2            RSpectra_0.16-0         
## [25] ifultools_2.0-5          tibble_2.1.3            
## [27] withr_2.1.2              lazyeval_0.2.2          
## [29] mime_0.7                 magrittr_1.5            
## [31] crayon_1.3.4             evaluate_0.14           
## [33] xml2_1.2.0               tools_3.6.3             
## [35] hms_0.5.0                matrixStats_0.54.0      
## [37] stringr_1.4.0            munsell_0.5.0           
## [39] compiler_3.6.3           rlang_0.4.0             
## [41] grid_3.6.3               rstudioapi_0.10         
## [43] igraph_1.2.5             labeling_0.3            
## [45] base64enc_0.1-3          rmarkdown_1.14          
## [47] gtable_0.3.0             rARPACK_0.11-0          
## [49] reshape2_1.4.3           R6_2.4.0                
## [51] gridExtra_2.3            dplyr_0.8.3             
## [53] zeallot_0.1.0            readr_1.3.1             
## [55] stringi_1.4.3            readMzXmlData_2.8.1     
## [57] parallel_3.6.3           Rcpp_1.0.1              
## [59] vctrs_0.2.0              readBrukerFlexData_1.8.5
## [61] splus2R_1.2-2            DEoptimR_1.0-8          
## [63] tidyselect_0.2.5         xfun_0.8  
 


 

 

 

 

 


 
 

 
 
